# Proprioceptive errors in the localization of hand landmarks: What can be learnt about the hand metric representation?

**DOI:** 10.1371/journal.pone.0236416

**Published:** 2020-07-31

**Authors:** Valeria Peviani, Gabriella Bottini

**Affiliations:** 1 Department of Neuroscience, Max Planck Institute for Empirical Aesthetics, Frankfurt am Main, Germany; 2 Department of Brain and Behavioural Sciences, University of Pavia, Pavia, Italy; 3 Cognitive Neuropsychology Center, ASST Grande Ospedale Metropolitano Niguarda, Milan, Italy; 4 NeuroMI, Milan Center for Neuroscience, Milan, Italy; Anglia Ruskin University, UNITED KINGDOM

## Abstract

Proprioception acquires a crucial role in estimating the configuration of our body segments in space when visual information is not available. Proprioceptive accuracy is assessed by asking participants to match the perceived position of an unseen body landmark through reaching movements. This task was also adopted to study the perceived hand structure by computing the relative distances between averaged proprioceptive judgments (hand Localization Task). However, the pattern of proprioceptive errors leading to the misperceived hand structure is unexplored. Here, we aimed to characterize this pattern across different hand landmarks, having different anatomo-physiological properties and cortical representations. Furthermore, we sought to describe the error consistency and its stability over time. To this purpose, we analyzed the proprioceptive errors of 43 healthy participants during the hand Localization Task. We found larger but more consistent errors for the fingertips compared to the knuckles, possibly due to poorer proprioceptive signal, compensated by other sources of spatial information. Furthermore, we found a shift (overlap effect) and a temporal drift of the hand perceived position towards the shoulder of origin, which was consistent within and between subjects. The overlap effect had a greater influence on lateral compared to medial landmarks, leading to the hand width overestimation. Our results are compatible with domain-general and body-specific spatial biases affecting the proprioceptive localization of the hand landmarks, thus the apparent hand structure misperception.

## Introduction

The central nervous system integrates visual and somatosensory information to represent the configuration of our body parts to navigate through space and interact with the environment. Specifically, the sense of proprioception provides somatic input that informs about the position and movement of body parts. The key receptors in proprioception are cutaneous stretch receptors and muscle and skeletal mechanoreceptors: muscle spindles, Golgi tendon organs, and joint capsule mechanoreceptors. These receptors encode postural, kinetic (e.g. force applied to a limb) and kinematic (e.g. velocity of the movement) information. This somatic information is thus essential to judge limb location, movement, force, heaviness, stiffness and viscosity, which makes proprioception closely tied to movement. Indeed, lesions involving the proprioceptive pathways may cause impaired coordination of voluntary movement, i.e., ataxia, characterized by symptoms that are usually worsened by the absence or paucity of visual cues. Proprioception is therefore crucial for motor control, from gait stability to coordination of fine hand movements, necessary for efficient object grasping and manipulation [1, 2]. As we are going to describe in detail here, the proprioceptive accuracy over the hand landmarks is characterized by a specific pattern of spatial biases, which may be relevant for the representation of our hand in space, as well as of the accuracy of fine motor control, grasping and manipulation.

To study the proprioceptive accuracy over a certain body landmark, researchers usually ask participants to match (without tactile feedback) the perceived position of that (unseen) body landmark through a reaching movement. Over the last decade, researchers have elegantly made use of this type of task (hereafter, the Localization Task, LT) to study the perceived structure of body parts (e.g. the hand [3]; the face [4]; the leg [5]). In the LT, participants are asked to localize the perceived position of a number of landmarks of a certain body part. The relative distances between the perceived locations of couples of landmarks are computed to extract the perceived dimensions of that body part. When the LT is used to study the hand structure, participants are required to point to the perceived locations of ten landmarks (fingertips and knuckles) of their occluded hand. In detail, while their hand lies under an opaque horizontal board, participants are asked to match the perceived position of each hand landmark with a stick held in the other hand. Typically, the perceived length of the fingers and the perceived hand width are obtained by computing the relative distance between the average perceived positions of couples of landmarks, and compared with the actual hand dimensions. Longo & Haggard’s seminal study [3], as well as later studies (e.g. [6–11]), reported a significant underestimation of the finger lengths and overestimation of the hand width, resulting in a disproportioned perceived hand shape.

While the perceived hand structure has been studied in detail across several conditions and populations (e.g. [7, 11, 12–20]), the spatial pattern of proprioceptive errors that leads to the misperceived hand structure remains unexplored. First, it is unknown how the proprioceptive errors vary across landmarks, and which landmarks are similarly mislocalized. Neuroimaging studies on humans showed that fingers are differently represented at the very early stages of tactile processing, namely in Brodmann Areas (BAs) 3b, 1 and 2 [21–23], and that these differences are reflected by behavioral measures, e.g. the tactile acuity [24]. Specifically, it has been reported that the increase in tactile acuity thresholds from the index to the little finger correlated with the decrease in cortical representation across the same fingers within the primary somatosensory cortex [24]. Two areas of the primate neocortex, BA 3a and 2, are believed to receive inputs from joint receptors and muscle spindles, and they are probably involved in proprioception [25–27]. The organization of BA 3a has been studied by performing multiunit electrophysiological recordings in macaque monkeys. It has been showed that this area contains a complete representation of deep receptors and musculature of the contralateral body, and that the general organization of body part representations mirrors the one of the primary somatosensory area, 3b [25]. It could be asked whether, similarly to what occurs in the tactile domain, proprioceptive localization accuracy varies across fingers in a way that resembles the cortical organization. Furthermore, it is acknowledged that proprioception is mostly mediated by muscle spindles [28, 29] and joint receptors [30], which are mechanoreceptors embedded in joints. It is therefore conceivable to hypothesize that the proprioceptive localization of the knuckles, which are joints surrounded by muscular tissue, would be more accurate than that of the fingertips. One of the aims of this study is therefore to investigate how the proximo-distal and medio-lateral components of the localization errors vary across the hand landmarks.

Second, it is unknown whether the localization errors of the hand landmarks are consistent within and between participants, or whether they are idiosyncratic, i.e. they largely vary across subjects. In regard of the proprioceptive localization of the hand, previous research [31] reported a consistent localization error. Researchers asked participants to perform movements with one hand (under a table) to match the position of the other hand (placed above the table, upon one out of nine locations placed on a horizontal line), while blindfolded. They found that the hand to be matched was consistently perceived to be shifted towards the shoulder of origin. However, when a different spatial pattern of locations was used (e.g. a grid of 48 possible locations [32]; or six locations in a 2D structure [33]), idiosyncratic errors were described for proprioceptive localization judgments. Here, we aimed at studying the consistency of the errors within and between subjects in the proprioceptive localization of several hand landmarks. Specifically, we focused on the consistency of the error direction (angle consistency), measured as the inter-trial phase clustering (ITPC) of the error vectors within and between subjects.

Finally, we decided to extend our investigation to the temporal characterization of the error pattern, instead of limiting it to its spatial features. The LT is suitable to test whether the localization errors are overall stable during the task, or instead increase or decrease over trials, providing insights on the consistency of proprioceptive localization over time. To this purpose, we included the trial number as a predictor in our analyses on the error components and angle consistency.

To resume, this study set out to characterize the spatial pattern of the errors pertaining to the proprioceptive localization of hand landmarks, through the administration of the LT. Concretely, we present a detailed investigation specifically focused on: i) the variation of the proximo-distal and medio-lateral error components across hand landmarks and over trials, ii) the consistency of the error direction within- and between- subjects and its variation across landmarks and over trials. We believe that the characterization of the pattern of proprioceptive mislocalization of the hand landmarks in the LT would provide relevant insights into the mechanisms underlying the apparent hand structure misperception quantified in previous studies.

For each LT trial, i.e., judgement of the perceived position of a certain hand landmark, we computed the systematic error, which represents the mismatch between the actual and perceived location of a certain landmark, and can be represented as a vector linking the actual landmark location to the perceived landmark location. Each error vector can be described in terms of magnitude, which can be decomposed in its medio-lateral and proximo-distal components, and in terms of direction, which can be expressed in degrees (the MATLAB [34] based code for these and other spatial preprocessing steps, along with data visualization functions, is freely available [35]). In order to conduct this investigation, we pooled the datasets of two experiments, in which participants performed the LT following established procedures [3].

## Methods

### Participants

The first dataset, extracted from an experiment aimed at investigating the role of the hand representation on simple movement [11], included 21 healthy participants, who performed the LT before or after a Proprioceptive Matching Task. Since we demonstrated that performing the Proprioceptive Matching Task did not influence the hand misestimation when measured afterwards through the LT [36], we included the entire sample, regardless of the task order.

The second dataset, extracted from an experiment aimed at investigating the effect of sensorimotor information on the hand map distortions [36], included 22 healthy participants, who performed the LT as a baseline measurement, before undergoing the Proprioceptive Matching Task or a control condition.

Participants (32 F, 11 M) were aged 20 to 39, with mean age 23.14 ± 3.80 years. Their average hand dimensions were 7.526 ± 0.633 cm (mean finger length, with each finger length measured from its fingertip to the knuckle) and 6.039 ± 0.509 cm (hand width, as the distance between the index and little knuckles). All the participants were right-handed, as confirmed by the administration of the Edinburgh Handedness Inventory [37] (mean 76.44 ± 32.11). Standard deviations have been reported as measures of dispersion. Participants gave their informed consent before the experiments and received university credits in return. The study received approval from the local ethical committee (University of Pavia) and adhered to the ethical standards of the Declaration of Helsinki.

### Procedure

The LT was administered following established procedures [3]. While seated in front of a table, participants were blindfolded and their left hand was placed in a white cardboard box (35 x 35 x 6 cm) fixed to the table. A baseline photograph (1280 × 960 pixels) was taken of the participant’s left hand, in position, before closing the box. The camera (Microsoft LifeCam VX-2000) was located 50 cm above the apparatus. The baseline photographs were used to extract coordinates of the hand actual landmarks. The photograph was compared with the hand position at the end of the experiment to ensure that no remarkable changes in finger configuration had occurred during the task. The blindfolding was removed once the box lid had been closed, to allow full visual feedback of the box. The first dataset includes 400 trials per participant, with 40 trials per each hand landmark administered in a random order. The second dataset, instead, includes 160 trials per participant, with 16 trials per each hand landmark administered in a random order. At the beginning of each trial, participants were instructed to localize the perceived location of a hand landmark (e.g. ‘Index fingertip’), by pointing with a stick, held in their right hand, over the box surface. After each judgement, a picture was taken for offline data processing and a new trial would begin.

### Analysis

#### Spatial preprocessing

For each participant, the X and Y coordinates (in pixels) of the actual hand landmarks were extracted from the baseline photographs. Then, for each trial, we extracted the X and Y coordinates (in pixels) of the tip of the stick pointing to the perceived landmark location. All the coordinates were re-centered on a common origin and transformed in cm by dividing the X and Y values for the participants’ conversion index (number of pixels per cm). Next, for each participant, we applied a rotation in order to align the little-index line (segment linking the little knuckle with the index knuckle) of the actual hand to the horizontal axis. Then, we applied the same rotation matrix to all the perceived and actual coordinates.

Afterwards, we detected and excluded multivariate outliers by computing the Mahalanobis distances and comparing them to a Chi-square distribution. Concretely, for each trial, we computed the Mahalanobis distance (in squared units) of the landmark perceived location from the average perceived location of that landmark. Next, for each landmark, we compared the Mahalanobis distance distribution against a Chi-square distribution with two degrees of freedom and excluded observations falling above the 95^th^ percentile.

**Fig 1:**
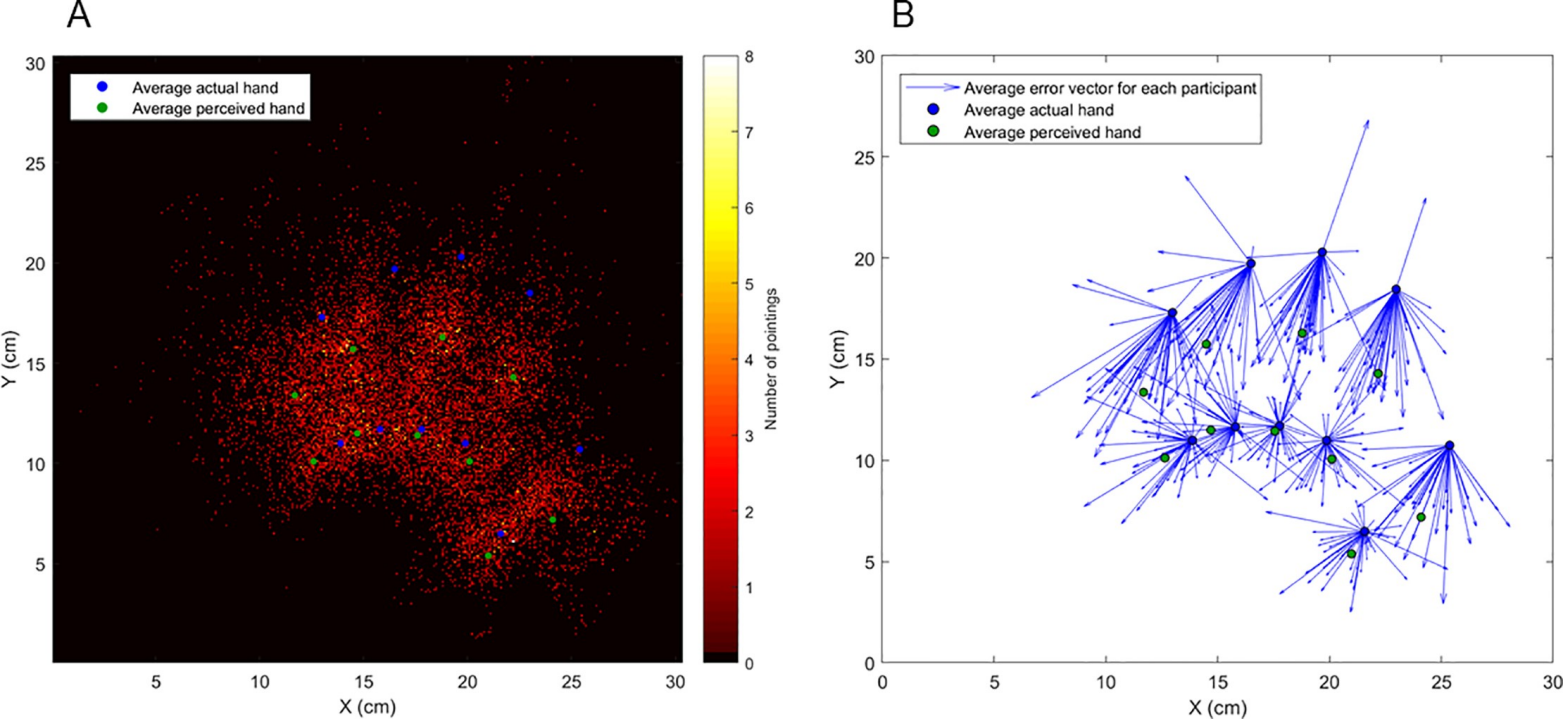
The localization errors starting from the average positions of the actual hand landmarks are plotted in a heatmap **(A)** and as vectors **(B)**. In both figures, blue dots represent the locations of the average actual landmarks, whereas green dots represent the locations of the average perceived landmarks.

For each trial, the vectors representing the systematic errors were computed. In detail, the systematic error was computed as the vector starting from the actual landmark location and ending to the perceived landmark location for that trial. The medio-lateral ($\vec{v_{x}}$) and proximo-distal ($\vec{v_{y}}$) components and the error direction (α) were extracted. The medio-lateral and proximo-distal components were computed as the projection of the vector onto the horizontal and vertical axes. Their signs reflect the direction of the error on the medio-lateral and proximo-distal axes: i) $\vec{v_{x}}$ < 0 describes a lateral error (toward the shoulder line); ii) $\vec{v_{x}}$ > 0 describes a medial error (toward the body midline); iii) $\vec{v_{y}}$ < 0 describes a proximal error (closer to the body); iv) $\vec{v_{y}}$ > 0 describes a distal error (farther from the body). The heat map in **Fig 1A**, depicts the ending points of the systematic error vectors of all the trials and participants, starting from the mean actual hand locations (in blue), showing the large variability of the judgments. **Fig 1B** represents the systematic error vectors, each averaged across trials, for each participant and landmark.

#### Statistical analyses

First, we aimed at studying the variation of the error components (proximo-distal and medio-lateral), across landmarks and trials. Since the datasets included a different number of trials, we normalized the trial number by dividing it by the total number of trials (400 or 160) and multiplying by 100. Given that a different number of trials might have an influence on task performance, due to factors such as fatigue, learning and/or attentional drop, we ascertained that the effect of normalized trial on the error components and angle consistency did not differ between experiments (**S1 File**), and that our key results did not change substantially by constraining the analyses to the first 160 trials of each experiment. Afterwards, we fitted a Linear Mixed-Effects Model for each of the two dependent variables (medio-lateral and proximo-distal errors), with finger (five levels: thumb, index, middle, ring and little), type (two levels: fingertip and knuckle), and normalized trial as fixed effects, incorporating random intercepts and slopes to account for inter-subject variability. We then ascertained that the residuals did not violate the normality assumption. Next, we conducted post-hoc tests to investigate significant effects, and applied corrections for multiple comparisons (Bonferroni).

Second, we aimed at quantifying the consistency of the error direction within and between subjects, across landmarks and trials. We defined the error direction as the angle in degrees between the vector and the horizontal axis. For instance, α = 0° represents a perfectly medial error (its proximo-distal component is zero), α = 90° describes a perfectly distal error (its medio-lateral component is zero), whereas α = 70° represents an error whose medio-lateral and proximo-distal components are both greater than zero. As a measure of angle consistency, we used the inter-trial phase clustering (ITPC) index, which is often adopted in electrophysiology research to measure the phase-coherence of the signal at a certain time point across trials. We applied Euler formula to describe each trial’s observation as a complex number (by multiplying by the complex number *i*), thus as a complex vector ($\vec{r_{1}}$, having $\left|\vec{r_{1}}\right|$ = 1 and the same direction of that trial’s error (α in radians):

$$\vec{v}=e^{i*\alpha }$$

To compute the ITPC index, our measure of angle consistency, a certain subset of complex vectors (e.g. those pertaining to each participant; those pertaining to each landmark and trial; etc., see details below) were averaged, and the magnitude (absolute value) of the average vector was extracted (See **S3 Fig in S1 File**). The angle consistency is comprised between 0 and 1, where 1 represents an exact overlap among the error vectors considered.

$$\mathrm{Consistency}\;\mathrm{of}\;\mathrm{error}\;\mathrm{direction}\;(\mathrm{angle}\;\mathrm{consistency})=|\sum_{i=1}^{n}\vec{r_{i}}/n|$$

In detail, the overall within-subjects angle consistency was calculated by extracting the absolute value of the average of the complex vectors of each participant, regardless of the landmark, and by computing the median of the resulting distribution. The within-subjects angle consistency was also extracted for each participant and landmark, and entered in the analyses. Specifically, we fitted a Generalized Linear Model with Gamma distribution and log-link to investigate the effect of finger (five levels: thumb, index, middle, ring and little) and type (two levels: fingertip and knuckle) on the within-subjects angle consistency.

The overall between-subjects angle consistency was calculated by extracting the absolute value of the grand average of the complex vectors. For each participant, we also extracted the most frequent direction of their errors (hereafter, most frequent error direction) by computing the phase angle of their average complex vectors. The between-subjects angle consistency was also calculated for each landmark and cluster of normalized trials. In order to increase the reliability of these last estimates, i.e., to increase the number of datapoints for each estimated angle consistency value, the normalized trials in this analysis were grouped in 100 clusters (e.g. trials from 0.25 to 1 were clustered together). We fitted a Generalized Linear Model with Gamma distribution and log-link to investigate the effect of finger (five levels: thumb, index, middle, ring and little), type (two levels: fingertip and knuckle) and normalized trial cluster on the between-subjects angle consistency.

For these last analyses, medians, interquartile ranges and non-parametric statistics were adopted to describe and model the data since the within- and between- angle consistency distributions did not meet the normality assumptions.

## Results

**Fig 2:**
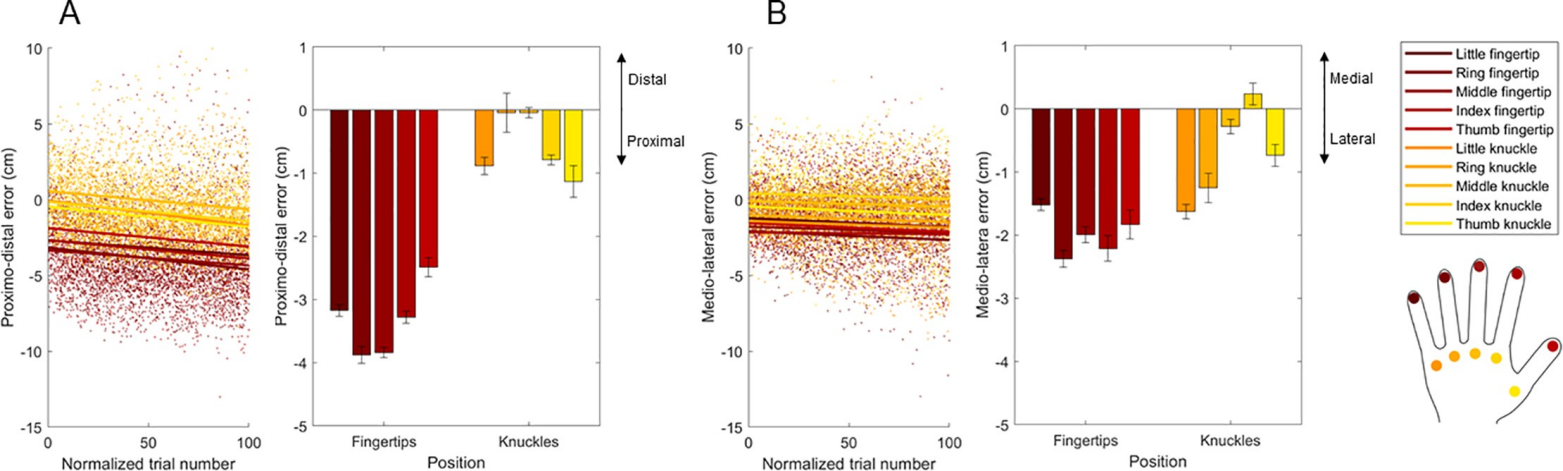
Slopes (left panels, scatterplots) obtained from the Linear Mixed-Effects Models describing the effect of normalized trial number on the proximo-distal **(A)** and medio-lateral **(B)** localization errors are represented for each hand landmark. Estimated marginal means (right panels, bar plots) obtained from the same models are represented for the proximo-distal **(A)** and medio-lateral **(B)** localization errors. Error bars indicate standard errors of the mean.

The first set of analyses aimed at investigating how the spatial components of the localization errors change over landmarks and trials. When the proximo-distal error component was considered as a dependent variable, the Linear Mixed-Effects Model detected a significant main effect of type (F(1,11333) = 502.060, p < .001), finger (F(4,11333) = 16.392, p < .001), normalized trial (F(1,1133) = 12.770, p < .001) and a significant finger-by-type interaction (F(4,11333) = 11.984, p < .001). The normalized trial did not significantly modulate the effect of type (type-by-normalized trial: F(1,11333) = 1.022, p = .312), finger (finger-by-normalized trial: F(4,11333) = 1.069, p = .369), nor their interaction (three-way interaction: F(4,11333) = .955, p = .431). The proximal component of the error similarly increased for all the landmarks, over trials (see **Fig 2A**, left panel). The post-hoc tests revealed a greater proximal error for the fingertips (-3.332 ± 0.111) compared to the knuckles (-0.577 ± 0.170). In detail, the proximal error was greater for each fingertip compared to the each knuckle, except for the thumb knuckle, for which the error was not different from that of the thumb, index and little fingertips, and the little knuckle, for which the error was not different from that of the thumb fingertip. After Bonferroni correction, the post-hoc tests did not reveal significant differences across fingers, regardless of landmark type (thumb: -1.811 ± 0.199; index: -2.0329 ± 0.087; middle: -1.943 ± 0.082; ring: -1.959 ± 0.220; little: -2.028 ± 0.115). Within the knuckles, we found a greater proximal error for the thumb, index and little knuckles, compared to the middle and ring ones. Within the fingertips, we detected a greater proximal error for the middle and ring fingertips compared to the other ones. See **Fig 2A**, right panel. Estimated marginal means and their standard errors have been reported in text or plotted in **Fig 2A**. The alpha-threshold for post-hoc tests was corrected for multiple comparisons (Bonferroni).

When the medio-lateral error component was considered as a dependent variable, the Linear Mixed-Effects Model detected a significant main effect of type (F(1,11333) = 65.798, p < .001) and finger (F(4,11333) = 24.818, p < .001), as well as a significant finger-by-type interaction (F(4,11333) = 16.905, p < .001). Instead, the main effect of normalized trial (F(1,11333) = 2.768, p = .096), the type-by-normalized trial interaction (F(1,11333) = .604, p = .437), the finger-by-normalized trial interaction (F(4,11333) = .635, p = .637) and the three-way interaction (F(4,11333) = 1.372, p = .241) were not significant (see **Fig 2B**). The post-hoc tests revealed greater lateral errors for the fingertips (-1.986 ± 0.156) compared to the knuckles (-0.732 ± 0.161). Coherently, we found a greater lateral error for each fingertip compared to each knuckle, except for the little finger knuckle, for which the error differed only from that of the ring fingertip, and for the ring finger knuckle, for which the error did not differ from that of the little fingertip. Furthermore, the post-hoc comparisons revealed a greater lateral error for the ring finger (-1.813 ± 0.220) compared to the other ones (thumb: -1.284 ± 0.200; index: -0.988 ± 0.188; middle -1.135 ± 0.119; little: -1.573 ± 0.115), and for the little finger compared to the index and middle fingers. We also detected differences within all the knuckles, with the greatest lateral error for the little knuckle, followed by the ring, thumb and middle ones, while the average error for the index knuckle was medial. Within the fingertips, a greater lateral error was detected for the ring (compared to all the other fingertips) and index fingertip (compared to the little and thumb fingertips). The proximal error for the middle fingertip was also greater than that for the little fingertip. See **Fig 2B**. Estimated marginal means and their standard errors are reported in text or plotted in **Fig 2B**. The alpha-threshold for post-hoc tests was corrected for multiple comparisons (Bonferroni).

We further investigated the pattern of increasing index-to-little lateral error through a Linear Mixed-Effects Model that considered the effect of the finger knuckle coded as a continuous variable (index to little: 1 to 4) on the medio-lateral errors. This analysis confirmed a significant linear effect of finger knuckle (F(1,5674) = 27.927, β = -0.318, p < .001). Such asymmetry in the medial errors for knuckle localization is likely to at least partially explain the hand width overestimation reported in previous studies (e.g. [3, 6, 10, 11, 15]). We confirmed this by modelling the random slopes obtained from the previous analysis as predictors of the hand width estimation ratio (ER). The hand width ER was obtained by computing the ratio between the actual and perceived hand width in cm. If the ER < 1, the dimension is underestimated, whereas, if the ER > 1, the dimension is overestimated. For each participant, the actual hand width was computed as the Euclidean distance between the actual little and finger knuckles, whereas the perceived hand width was computed as the Euclidean distance between the perceived positions (averaged across trials) of the little and index finger knuckles. The increasing lateral error from the index to the little knuckle explained about 80% of the hand width ER variance (adjusted R^2^ = 0.804, t(41) = -13.168, β = -0.635, p < .001). **See Fig 6A**.

Together, these results highlighted a different error pattern across landmarks, with a clear difference between fingertips and knuckles. Furthermore, we found that the proximo-distal component of the error linearly decreased over trials.

The second set of analyses focused on the within- and between-subjects consistency of the error direction. The angle consistency corresponds to the absolute value, i.e., the length, of the vector $\vec{r}$ representing the average of a certain subset of complex vectors ($\vec{r_{1}},\vec{r_{2}},\ldots ,\vec{r_{n}}$). For instance, the complex vectors pertaining to each participant have been used to compute the overall within-subjects angle consistency; the complex vectors pertaining to each landmark and trial, regardless of the participant, have been used to compute the between-subjects angle consistency; etc. (see Statistical analyses). This value ranges from 0 to 1, where 1 represents an exact overlap among the error vectors considered (See **S3 Fig** in **S1 File**).

**Fig 3:**
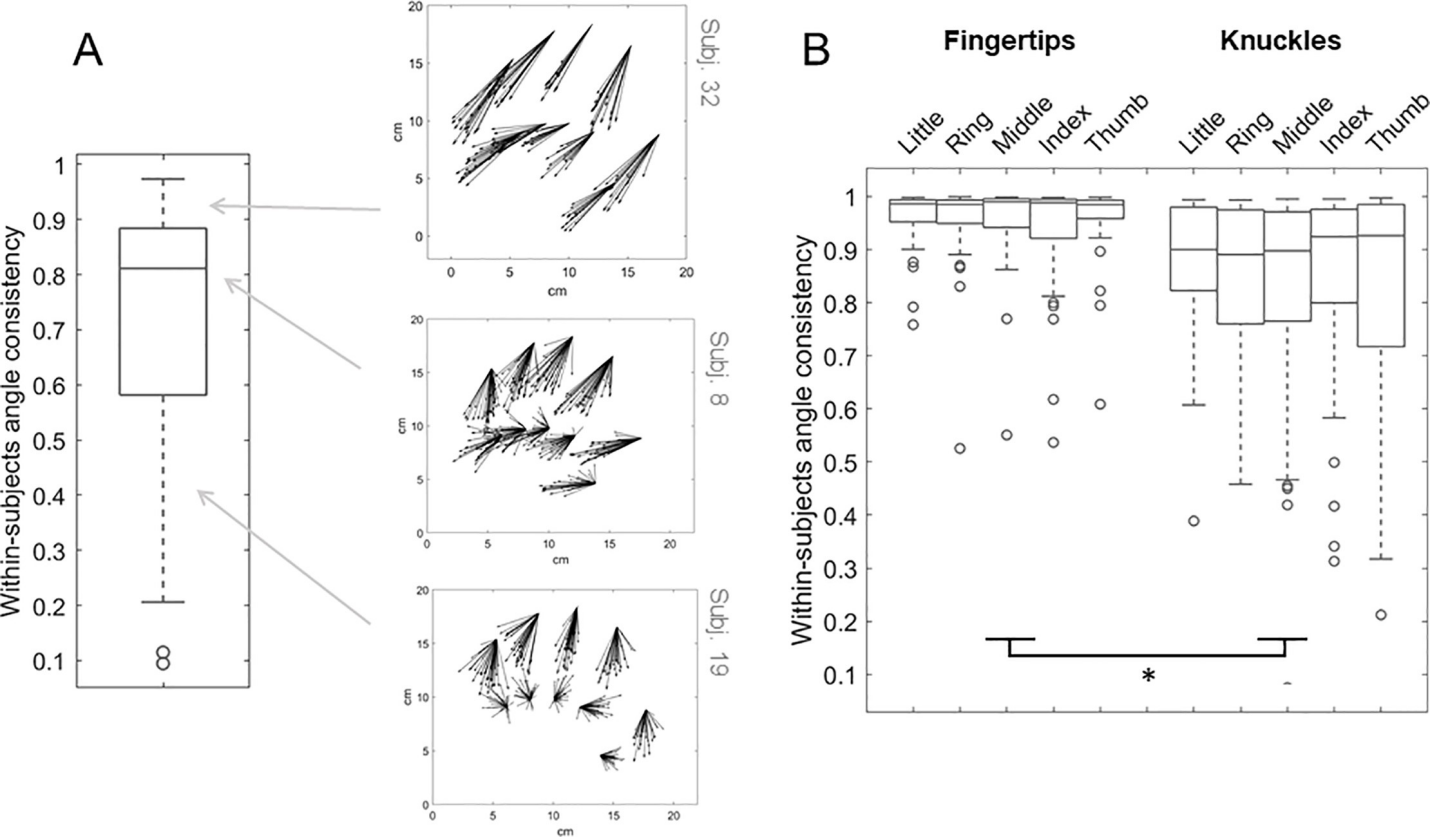
**A)** The distribution of the within-subjects angle consistency is represented in a box-and-whiskers plot. The error vectors of three exemplificative participants are depicted. **B)** The within-subjects angle consistency for each landmark is represented through box-and-whiskers plots. The angle consistency is significantly higher for fingertips compared to knuckles, as detected by the Generalized Linear Model (main effect of type) and signaled by the asterisk.

The overall within-subjects angle consistency, regardless of the landmark, was very high (median ± interquartile range: 0.811 ± 0.304, **Fig 3A**), meaning that each participant was highly consistent in the direction of his/her errors. When the landmark was considered, within-subjects angle consistency resulted greater for the fingertips (median ± interquartile range: 0.986 ± 0.051) compared to the knuckles (0.909 ± 0.190), as detected by the Generalized Linear Model (significant main effect of type: Wald χ^2^(1) = 42.278, p < .001, non-significant effect of finger: Wald χ^2^(4) = 1.913, p = .752; non-significant interaction: Wald χ^2^(4) = 1.439, p = 837). See **Fig 3B**.

**Fig 4:**
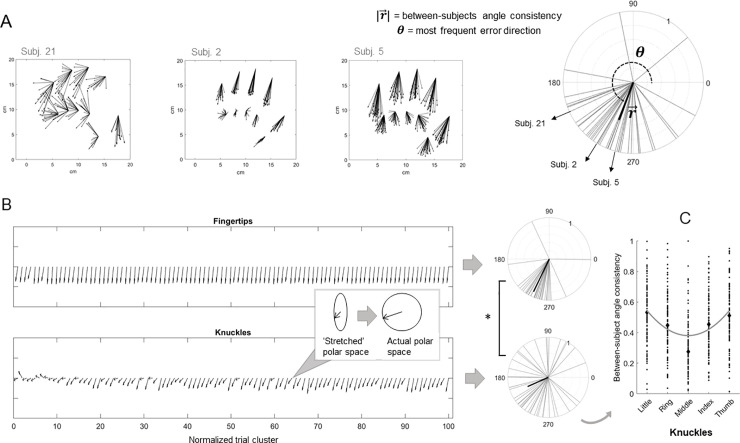
**A)** The between-subjects angle consistency is represented in a polar plot as the magnitude, i.e., the length, ranging from 0 to 1, of the vector $\vec{r}$ ($\left|\vec{r}\right|$), oriented towards the most frequent error direction (*θ*). The error vectors of three exemplificative participants are depicted. **B)** The between-subjects angle consistency did not significantly change over time but varied across fingertips and knuckles. To illustrate this, we plotted, for each normalized trial, the error direction separately for fingertips and knuckles, as a vector with constant magnitude 1 (note that, for visualization purposes, the proximo-distal dimension is ‘stretched’ compared to the medio-lateral one, therefore each vector is represented in an elliptical, instead of circular, polar space, as depicted in the box). The between-subjects angle consistency for fingertips and knuckles is represented in the polar plots aside, as the magnitude, i.e., length, ranging from 0 to 1, of the average error vector, oriented towards the most frequent error direction for each landmark type. The asterisk indicates the significant main effect of type **C)** The quadratic function describing the effect of finger knuckle (coded as 1 to 5) on the between-subjects angle consistency is plotted in gray. Black diamonds indicate medians.

The overall between-subjects angle consistency was moderately high (0.565), meaning that different participants made errors within a relatively small range of directions (most of them around a median of 241.369 ± 39.336°, see **Fig 4A**). Furthermore, the between-subjects angle consistency varied across landmarks, and was higher for the fingertips compared to the knuckles, but did not vary across trials (**Fig 4B**). Indeed, the Generalized Linear Model investigating the effect of finger, type and normalized trial cluster on the between-subjects angle consistency detected a significantly higher angle consistency for the fingertips (median ± interquartile range: 0.874 ± 0.140) compared to the knuckles (0.458 ± 0.330), as detected by the significant main effect of type (Wald χ^2^(1) = 202.650, p < .001), represented in **Fig 4B**. The effect of finger was also significant (Wald χ^2^(4) = 11.221, p = .024), with greater angle consistency values for the thumb (0.766 ± 0.350) compared to the other fingers (index: 0.716 ± 0.441; middle: 0.642 ± 0.620; ring: 0.729 ± 0.440; little: 0.704 ± 0.350) and lower values for the middle finger compared to the other fingers, as detected by Bonferroni-corrected post-hoc comparisons. The main effect of finger seems to be driven by differences across knuckles, with the thumb (0.533 ± 0.273) and little (0.513 ± 0.274) knuckles showing greater angle consistency, and the middle finger knuckle (0.2757 ± 0.222) showing lower angle consistency, compared to the other knuckles (index: 0.448 ± 0.231; ring: 0.455 ± 0.310), as detected by the finger-by-type interaction (Wald χ^2^(4) = 22.062, p < .001) and Bonferroni-corrected post-hoc comparisons. Instead, no differences were detected across fingertips. A quadratic function with finger knuckle modeled as a continuous variable (1 to 5) and the between-subjects angle consistency as the dependent variable (linear effect of finger: t(2, 497) = -7.519, β = -0.245, p < .001; quadratic effect of finger: t(2,497) = 7.679, β = 0.041 p < .001) well described the pattern of angle consistency across finger knuckles (**Fig 4C**). This suggests that the consistency of the error direction across participants decreases from the hand medial and lateral boundaries to the hand center. No other significant effects were found (normalized trial cluster: Wald χ^2^(1) = 1.819, p = .177; type-by-normalized trial cluster: Wald χ^2^(1) = 1.159, p = .282; finger-by-normalized trial cluster: Wald χ^2^(4) = 6.637, p = .156, three-way interaction: Wald χ^2^(4) = 1.163, p = .884).

**Fig 5:**
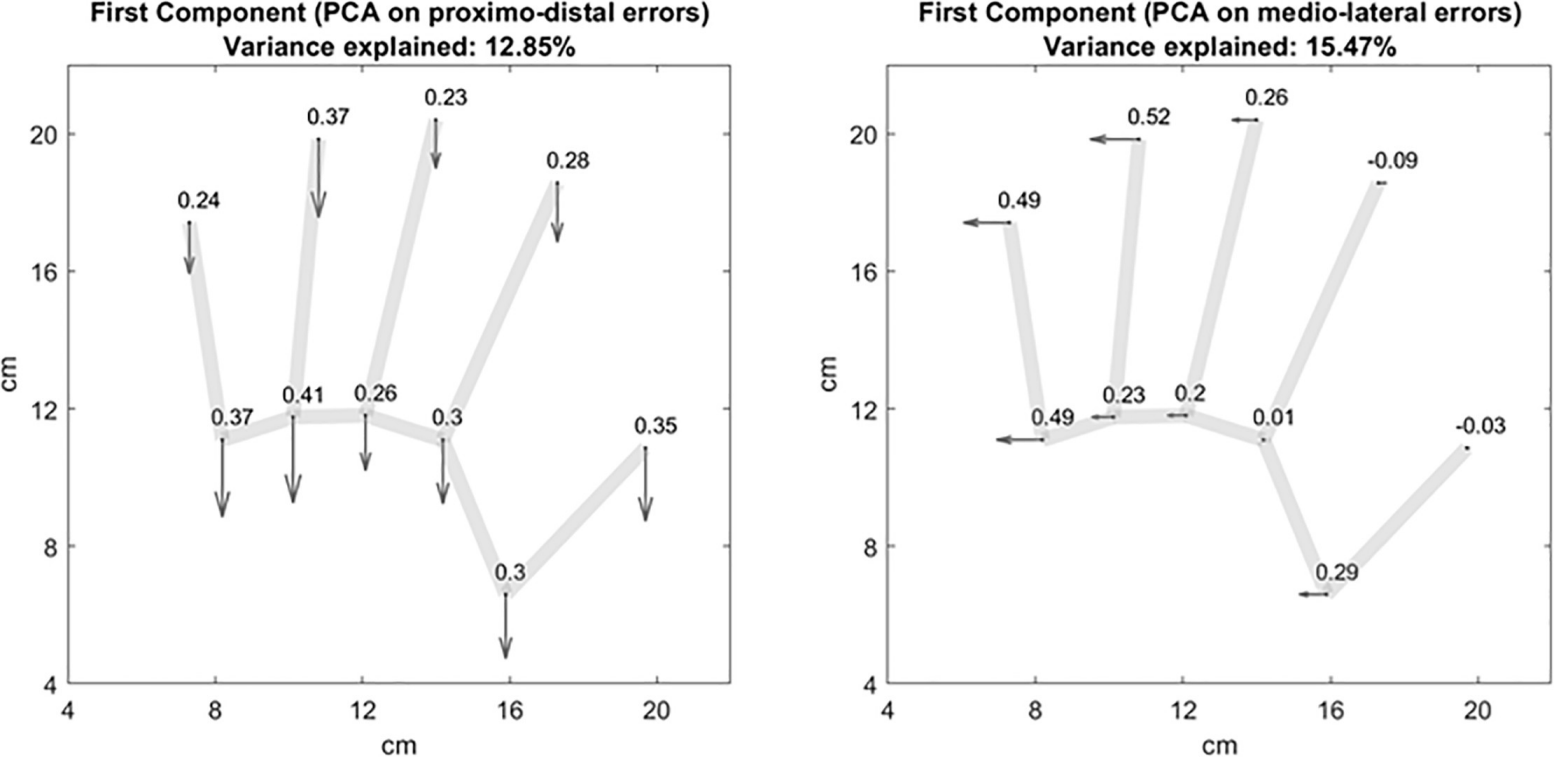
**The PC1s (first principal components) of the PCAs on proximo-distal (left panel) and medio-lateral (right panel) errors are represented starting from the average positions of the actual hand landmarks.** For each hand landmark, the average error of the PC1 is represented as a vector along with its loading.

**Fig 6:**
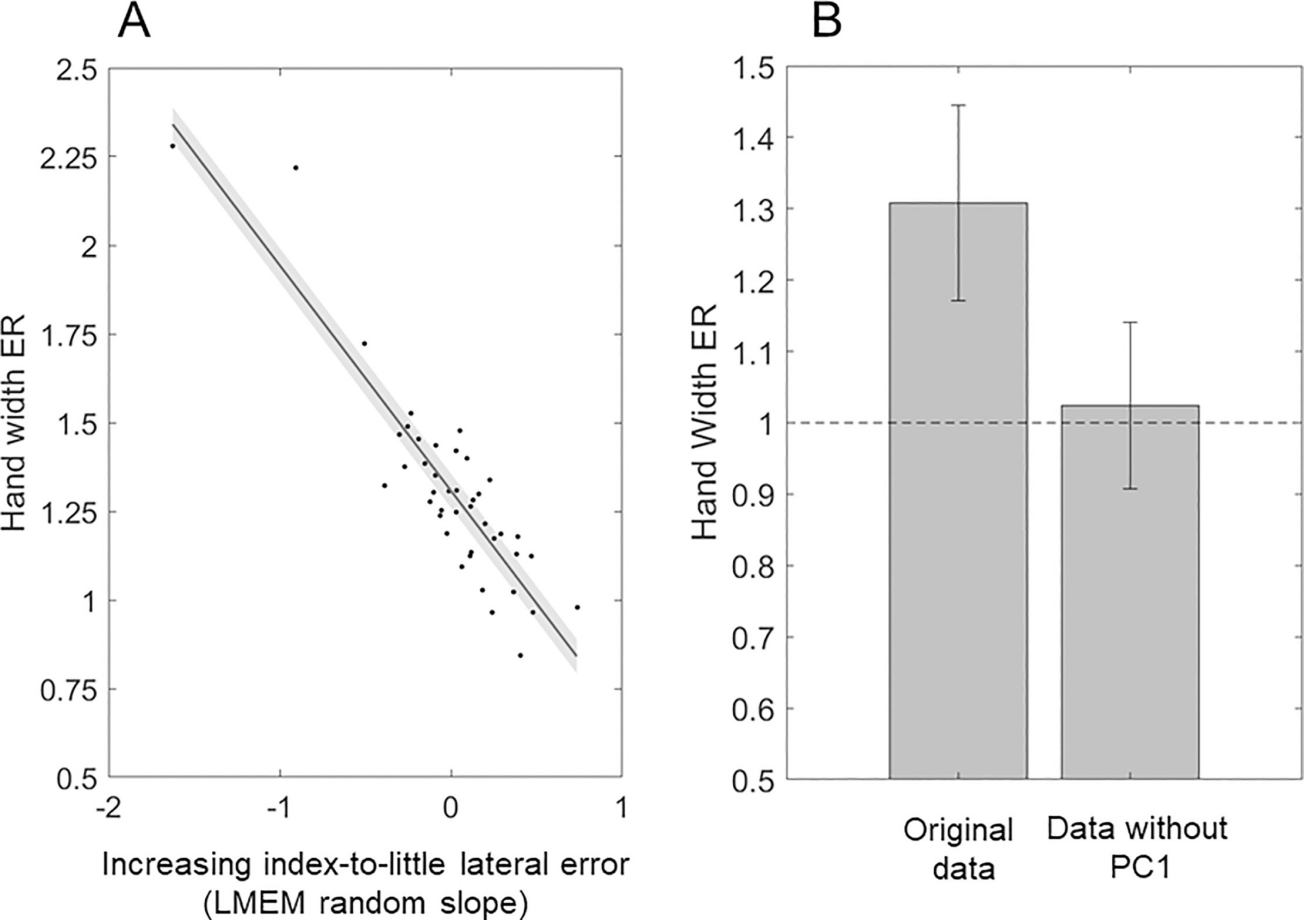
**(A)** The slopes obtained from the Linear Mixed-Effects Model (LMEM) exploring the effect of the knuckle (coded as a continuous variable, 1 to 4) on the medio-lateral errors, which strongly predicts the hand width estimation ratio (hand width ER: perceived / actual hand width). The shaded area represents the slope’s standard error of the mean. **(B)** The hand width ER calculated on the original data is higher than the hand width ER calculated on the same data after removing the PC1 (first principal component) extracted by performing the PCA on the medio-lateral errors.

From these last results and raw individual plots it seems that, at least for many participants, there is a source of error that is similarly represented across landmarks, which is responsible for the high angle consistency within and between subjects. In order to test for the presence of this shared error source, we performed a Principal Component Analysis (PCA) on the proximo-distal and medio-lateral errors. PCA allows the identification of weighed combinations of the original variables (components) that better explain the total variability of the data. In other words, this technique captures the main—orthogonal -sources of variation in the data. The PCA on the proximo-distal errors well captured a shared source of proximal error in the first principal component (PC1), i.e., the component that accounts for the greatest amount of variability in the data. The loadings of a principal component indicate how much each variable ‘loads on’, or contribute to, that component. The magnitude and sign of a component’s loadings provide information about the influence of that component on the variables considered. The loadings of the proximo-distal PC1 were overall similar across landmarks, with slightly higher values for the knuckles (average loading for the knuckles: 0.328 ± 0.057) compared to fingertips (0.294 ± 0.062). See **Fig 5**. The PC1 extracted from the PCA on the medio-lateral errors also seems to reflect a common source of lateral error, albeit not homogenously affecting all the landmarks. Indeed, for the medio-lateral PC1 we observed increasing loadings from the index to the little finger, regardless of the landmark type (thumb: 0.130 ± 0.226; index: -0.037 ± 0.071; middle: 0.229 ± 0.046; ring: 0.375 ± 0.204; little: 0.488 ± 0.001). The medio-lateral PC1 therefore describes a source of error that has a greater influence on lateral, compared to medial, landmarks. This index-to-little loading increase is likely to underlie the index-to-little lateral error increase reported observed for the knuckles (**Fig 2B**), which turned out to be strongly predictive of the overestimation of the hand width (**Fig 6A**). As a matter of fact, once the medio-lateral PC1 had been removed from the data, the hand width overestimation was minimized, i.e., the hand width ER was significantly smaller than the original hand width ER (t(42) = 11.759, p < .001) and not significantly different from one (t(42) = 0.676, p = .234). See **Fig 6B**.

Key results are resumed in **Table 1**. The spatial preprocessing and statistical analyses were performed in MATLAB [34]. The MATLAB code for data preprocessing and visualization is freely accessible (Peviani, 2019).

**Table 1:** Summary of results.

Variable	Fingertips vs. knuckles	Across fingertips	Across knuckles	Over trials
**Proximo-distal error (Fig 2A)**	Proximal: fingertips > knuckles	Proximal: middle and ring > index and little > thumb	Proximal: Little, index and thumb > middle and index	Proximal drift over trials, similar across landmarks
**Medio-lateral errors (Fig 2B)**	Lateral: fingertips > knuckles	Lateral: ring and index > middle and thumb > little	Lateral: little > ring > thumb > middle > index (medial)	No significant change over trial
**Within-subjects angle consistency (Fig 3B)**	Fingertips > knuckles	No differences	No differences	/
**Between-subjects angle consistency (Fig 4B)**	Fingertips > knuckles	No differences	Decreases from the hand boundaries to the center	No significant change over trial

For each variable considered in the analyses (proximo-distal and medio-lateral error components, within- and between-subjects angle consistency) the key results regarding differences across landmark type (fingertips vs. knuckles), across fingertips, across knuckles and over trials are resumed. Note that differences across fingertips and knuckles in terms of proximo-distal and medio-lateral components are indicative. See the main text for more detailed information.

## Discussion

When visual feedback is not available, proprioceptive inflow is the main source of information the central nervous system relies on to localize a body part. Proprioceptive accuracy is therefore assessed by asking participants to match the perceived position of a body landmark through a reaching movement. Recently, researchers have been using this type of task to study the perceived structure of the hand as well as other body parts [3–5]. In the hand LT, participants are required to point to the perceived location of specific landmarks (fingertips and knuckles) of their occluded hand. The relative distances between the average perceived positions of couples of landmarks are computed and compared with the actual hand dimensions. The LT thus provided interesting insights about how we apparently (mis)perceive our hand structure. Nevertheless, the proprioceptive accuracy of hand landmark localization is still unknown. Here, we set out to explore the spatial and temporal features of the proprioceptive error pattern arising from the LT administration to 43 healthy participants. Specifically, we analyzed: i) the variation of the proximo-distal and medio-lateral error components across hand landmarks and over trials, ii) the consistency of the error direction (angle consistency) within and between subjects and its variation across landmarks and over trials. We believe that analyzing the pattern of proprioceptive localization errors for the hand landmarks may provide relevant insights into the origin of the apparent hand structure misperception quantified in previous studies (e.g. [3, 6, 10, 11, 15, 17]).

Overall, we found that the localization accuracy varies across hand landmarks, especially between landmark types (fingertips vs. knuckles). In detail, the magnitude of proprioceptive errors is generally larger, with greater proximal and lateral components, for the fingertips compared to the knuckles (**Figs 1** and **2**). The different patterns of errors might reflect a difference in terms of anatomical and physiological features of fingertips and knuckles. First, knuckles are joints (metacarpophalangeal joints), while fingertips are not, despite they are very close to distal interphalangeal joints. As mentioned in the introduction, mechanoceptors located in the joint capsules respond to joint movements, contributing to kinesthesia, i.e., the sense of movement, as part of proprioception. Their contribution to position sense, also part of proprioception, is however limited, since they have a high mechanical threshold and mainly respond to the application of pressure, flexion, extension and rotation [29, 38]. Muscle spindles, instead, inform both movement and position sense. In particular, the primary and, even more, the secondary endings of muscle spindles have static length sensitivity, and they are considered the major proprioceptive sensors [28, 29]. Relatedly, knuckles differ from the fingertips in terms of another important aspect, which is the large amount of muscular tissue that surrounds them. It could be therefore hypothesized that the afferent somatic input informing fingertip proprioception may be poorer and/or noisier compared to that informing knuckle proprioception, at least in a task that does not involve movement, such as the LT. The role that this asymmetry, i.e., the different proprioceptive accuracy for fingertips and knuckles, plays on hand movements is worth being investigated further. While it seems unlikely that the poor accuracy in fingertip localization would affect the transport component of reach-to-grasp movements, which relies on the perceived position of the whole hand in space [39], it would be more interesting to investigate its effect on the grasp component (e.g. finger tuning and aperture), for which the perceived position of the fingertips might be more relevant.

Interestingly, we showed that the localization of fingertips and knuckles varied in terms of angle consistency as well: the between- and within-subjects consistency of the error direction is higher for the fingertips compared to the knuckles (**Figs 3B** and **4B**). Participants therefore made larger but more consistent errors when localizing the fingertips, with shifts that were systematically oriented towards the proximal and lateral directions. Considered alone, a noisier proprioceptive signal coming from a specific body landmark would predict larger but not necessarily more consistent errors in the localization of that body landmark. Therefore, we argue that the localization of the fingertips does not uniquely reflect a very noisy proprioceptive signal, instead, it may rely on the integration of additional sources of information. One possibility is that a prototype bias occurred during the localization of the fingertips. The prototype bias is a localization bias towards the center and within the boundaries of the categorical (or prototypical) space, which has been described both in spatial and tactile localization. The prototype, in this context, is indeed defined as ‘a central value in the category’ [40]. It has been proposed that remembering locations in space involves fine-grained information (based on estimates of the actual position of the stimulus) and categorical information [40]. Accordingly, when the fine-grained information is poor, localization judgments rely more on categorical information. For example, it has been shown [40] that, when remembering the location of a dot in a circle, participants made use of fine-grained information (polar angle of the remembered stimulus) and categorical information (quadrant to which the remembered stimulus ‘belongs’). Indeed, participants imposed horizontal and vertical boundaries that divide the circle into quadrants and misplaced dots towards the center of each quadrant and within the boundaries. As predicted, the prototype bias increased when the mnestic precision for a particular location was decreased by presenting a distractor task. Crucially, the prototype bias is functionally relevant, since it increases the overall accuracy of estimation. More recently, it has been pointed out that errors possibly reflecting prototype biases have been observed in tactile localization tasks administered to healthy individuals and patients with somatosensory damage [41]. Specifically, the prototype bias increased in conditions of increased somatosensory noise, such as low stimulus intensity or somatosensory damage. Healthy participants were asked to localize the location of tactile stimuli of varying intensity presented on the forearm [42]. As the stimulus intensity decreased, the bias towards the center of the forearm increased. Previous work also described two patients with left-hemispheric lesions involving the hand area in the primary somatosensory cortex who localized finger tactile stimuli towards the center of the (contralesional) hand [43].

Similarly, in the hand LT, the localization of the fingertips, for which the proprioceptive signal may be poorer compared to the knuckles, might have relied more on categorical information (e.g. the prototype might be the body part to which the fingertip ‘belongs’; thus the hand or the finger itself). Accordingly, most of the participants consistently misplaced the fingertips proximally, towards the center of the categorical space, which could be the hand, or the finger itself. This misplacement might have contributed to minimize errors in a condition of poor proprioceptive signal. If this is the case, the underestimation of the finger length reported in previous study might be explained by a general spatial bias which is shared with other modalities, and not by a misperceived hand representation. However, a smaller albeit significant underestimation of the perceived finger length has been reported in visual-based body representation tasks as well, which did not involve proprioceptive localization. For instance, in a recent study [44] we asked participants to compare the perceived dimensions of their unseen hand with the perceived length of line segments presented on a computer screen, using a two-alternative forced choice task. The finger length was underestimated on average, suggesting that the finger length underestimation observed in the LT does not uniquely reflect a domain-general spatial bias, such as the prototype bias. Rather, it is likely to reflect a body-specific metric bias, i.e., pertaining to the representation of the body, which might be combined with domain-general biases affecting the spatial judgements in the LT.

Another interesting result is that the consistency of the error direction between- (and not within-) subjects increased from the center to the boundaries of the hand dorsum, i.e., from the middle knuckle to the more lateral/medial knuckles (**Fig 4C**). In other words, participants’ errors were more similar in their direction when they judged the location of landmarks positioned close to the boundaries, compared to the center, of the hand dorsum. The fact that such pattern of results is not mirrored by a similar pattern regarding the within-subjects angle consistency is crucial for its interpretation. In detail, this means that, while for each participant the mislocalization of the middle finger knuckle is as consistent as that of the thumb knuckle, across participants this is not the case. The localization errors for the middle finger knuckle across different participants took a larger range of directions compared to those pertaining, for instance, to the thumb knuckle. We argue that participants may have relied more on structural information about the hand, such as the fact that the thumb knuckle is next to the hand physical boundary, to make localization judgments for the more lateral/medial knuckles. This type of knowledge, which is not conveyed by any sensorial cues, pertains to a long-term explicit component of body representation, namely the body structural description [45, 46]. This body representation informs on spatial relations of the body *in general*. While performing the LT, participants *know* that the thumb knuckle is next to the physical boundary of the hand, and might rely on this knowledge when making localization judgments, for example by avoiding too medial (or too lateral, when the LT assesses the right hand) errors. If participants rely on a general source of knowledge, their errors would be more similar. This structural knowledge might instead weigh less on the localization of knuckles that are further from the physical boundaries of the hand, for which the inter-individual variation of the error direction is indeed higher. The role of structural information on the LT judgements might be also salient for the localization of the fingertips, which are misperceived proximally due to large and consistent biases within the known boundaries of the hand.

Our analyses also showed differences in proprioceptive accuracy across fingers. However, these differences do not systematically mirror a pattern of increasing cortical representation from the little to the index finger, as for example reported by previous work [24] in tactile detection. Such pattern was only detected for the medio-lateral error component of knuckle localization, which decreased from the little to the index finger. Importantly, this pattern of increasing index-to-little lateral error turned out to be the major factor underlying the overestimation of the perceived hand width, explaining 80% of the hand ER variance (**Fig 6A**). Furthermore, such error pattern seems to be underlined by the greater weight of a major source of error on lateral compared to medial landmarks. This major source of error is represented by the PC1 extracted by the PCA on the medio-lateral errors. Its loadings increased from the index to the little finger, suggesting that this source of error explained a larger amount of variance pertaining to the localization of lateral landmarks, compared to medial ones. Importantly, this asymmetric bias was not specific to the knuckles, since it affected the fingertips as well (**Fig 5**). Once this source of error had been removed from the data, the hand width overestimation was minimized (**Fig 6B**). As we will discuss in the next paragraph, this source of error is likely to reflect the proprioceptive misplacement of the hand towards the shoulder of origin, known as the overlap bias [47, 48]. The hand width overestimation, which results from the pattern of increasing index-to-little lateral error, might therefore be a consequence of a greater overlap bias affecting the lateral compared to the medial landmarks.

So far, we have mainly discussed the relative differences between error components and consistency across hand landmarks. By considering the absolute direction of these errors, we found a strong tendency to mislocalize the position of all the landmarks towards the proximal and lateral directions. The within-subjects angle consistency, i.e. how consistent the direction of the error was within the performance of each participant, was indeed very high (**Fig 3A**), suggesting that there may be a source of bias which is similar across the localization judgments of each participant. This source of bias seems to be quite similar across participants as well, since the between-subjects angle consistency was moderately high (**Fig 4A**), with most participants making an average error within the third quadrant of the polar space (between 180° and 270°). This observation was supported by the PCA, which detected a shared source of proximal error in the PC1, whose loadings are overall similar across landmarks. The PC1 extracted from the PCA on the medio-lateral errors also seems to reflect a common source of lateral error, although, as previously mentioned, its effect is not homogeneous across different landmarks. Its weights are indeed greater for lateral, compared to medial landmarks, regardless of the landmark type (fingertip or knuckle). See **Fig 5**. Crucially, the fact that the bias similarly affected both fingertips and knuckles suggests that it is not specific to one type of landmarks, but affected the hand as a whole. We argue that this shift is likely to represent a proprioceptive misplacement of the hand toward the shoulder of origin, known as the overlap effect, and described in previous investigations [47, 48]. This effect was shown to have a perceptual, rather than a motor, origin, since it was minimized when the target (the hand to be matched) was visible [47]. This representational shift might be functional to object manipulation. When reaching for an object located in close to the center of our workspace, as it is often the case, it may be advantageous to overshoot with the limb (i.e., to overlap with the target) and rely on tactile feedback, rather than to undershoot the target and perform additional corrective movements [48]. The overlap effect is also compatible with the hypothesis that each hand operates in its egocentric space and uses a frame of reference based on its motor workspace, which is shifted towards the shoulder of origin [47]. By using different patterns of possible spatial locations for the target (the hand to be matched), previous studies detected idiosyncratic errors [32, 33]. In the future, it would be worth investigating whether the between-subjects angle consistency would decrease towards idiosyncrasy if the hand LT is performed in different spatial locations within the peripersonal space. Furthermore, the proprioceptive errors for the hand position estimation varies over the peripersonal workspace [32]. How (and if) this variation is modulated by the spatial position of the landmark itself (little finger vs. middle finger) or the type of the landmark considered (fingertip vs. knuckle) would inform about the weight of domain-general biases in the hand LT. Finally, it is interesting to note that the loadings of the PC1 extracted from the proximo-distal errors, possibly representing such proprioceptive misplacement, were slightly higher for the knuckles compared to the fingertips. This means that the source of bias reflected by the PC1 affected knuckle localization more than fingertip localization, on which other sources of bias, such as the prototype bias and finger length underestimation, might have had a greater influence.

When we considered the temporal features of the localization errors, we found that, while they did not significantly change over trials in the medio-lateral axis, their magnitude increased in the proximo-distal axis (**Fig 2**). Importantly, this change was similar for all the hand landmarks, meaning that it did not affect the perceived structure of the hand, rather it represents a drift of its perceived position towards the body over the course of the experiment. This drift has been documented in proprioceptive matching tasks (e.g. [49, 50], but see [51]) and at least partially explained as the effect of adaptation in discharge levels of sensory receptors in joint muscles [52]. Finally, between-subjects angle consistency did not significantly change over trials, suggesting that the source of bias similarly affecting localization judgments across participants is stable over time (**Fig 4B**).

### Relations with past research and future directions

An important venue for future research is testing whether and how the magnitude, direction and/or angle consistency of the localization biases are sensitive to variations of the experimental conditions. This could provide important insights on the nature of the metric biases affecting the hand representation.

**Fig 7:**
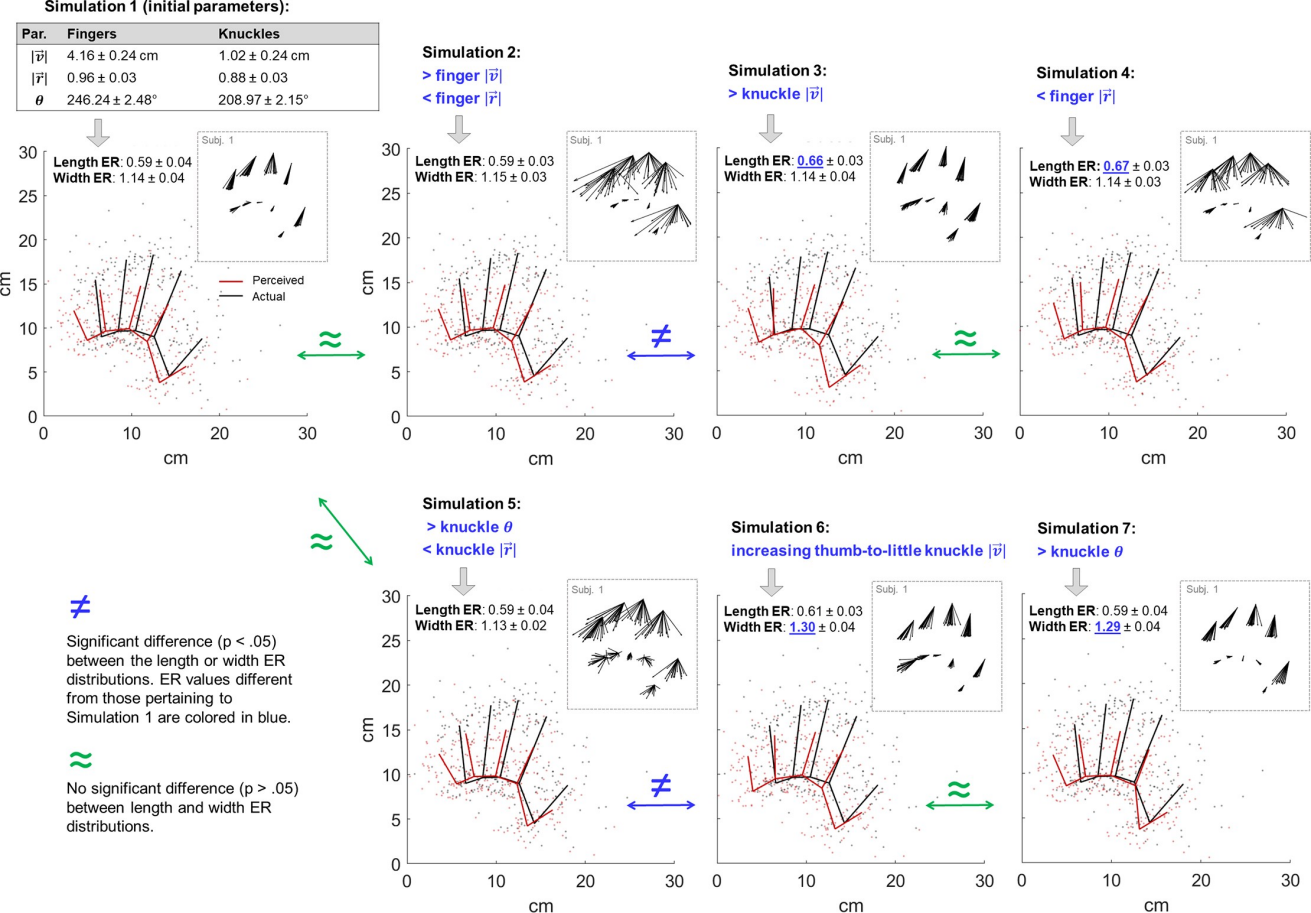
Simulations of seven patterns of perceived hand structure obtained by varying parameters of localization errors. The initial parameters, extracted from our real data, are reported in the top-left table ($\left|\vec{v}\right|$: magnitude of the average error vector; $\left|\vec{r}\right|$: angle consistency; *θ*: most frequent error direction). The perceived hand structure of Simulation 1 (top-left plot, red map) is obtained by multiplying the initial parameters of each landmark and adding inter-individual, -landmark and -trial noise (see **S1 File**). The resulting perceived hand structure is characterized by an underestimation of the mean finger length (length estimation ratio, ER: perceived / actual mean finger length), and an overestimation of the hand width (width ER: perceived / actual hand width). The error vectors of a representative subject are plotted inside the dashed frame. Simulation 2, 3, 4, 5, 6, and 7 are obtained by varying—of a similar intensity—one or more of the initial parameters (see **S1 File**). Similar (simulations 1 and 2; 3 and 4; 1 and 5; 6 and 7) and different perceived hand structures, in terms of mean finger length ER (1 and 3, 4; 2 and 3, 4) and hand width (1 and 6, 7; 5 and 6, 7), can be underlined by very different patterns of localization errors. ER distributions have been compared through parametric statistics (**S1 File**).

The perceived structure of the hand has been studied using the LT across several conditions (e.g. sighted vs. blindfolded [7]; verbal vs. tactile instructions [15]; real vs. imagined hand [12]) and populations (e.g. naïve vs. professional baseball players [17], naïve vs. magicians [14], males and females [20], healthy participants vs. an amputee [16]). Variations in the pattern of the hand perceived structure might be better understood by exploring the underlying variations in terms of localization biases. To provide some examples that illustrate this, we simulated a few datasets in which the magnitude, angle consistency and/or direction of the localization errors were manipulated, and compared the resulting patterns of perceived hand structures in terms of finger length and hand width (**Fig 7**). The procedure is described in detail in the **S1 File**. For instance, a reduced finger length underestimation, i.e., more accurate finger length estimation in condition B compared to condition A (or population B compared to population A) may be related to greater knuckle localization errors, such as in Simulation 3 vs. Simulation 1 (**Fig 7**), or to a reduced fingertip angle consistency, such as in Simulation 4 vs. 1 (**Fig 7**). As another example, a greater hand width overestimation may be related to greater knuckle errors, such as in Simulation 6 vs. 1 (**Fig 7**), or to different knuckle error directions, such as in Simulation 7 vs. 1 (**Fig 7**). On the other side, apparent similarity in the perceived hand structure across conditions or populations could also mask subtler differences pertaining to localization biases (e.g. Simulation 1 vs. 2, 3 vs. to 4, 1 vs. 5 and 6 vs. 7). In some cases, understanding the variations of the perceived hand structure across experimental conditions in terms of localization biases could be highly informative about the sources of information and thus of error contributing to the widely-reported distortions in the perceived hand structure.

For instance, previous studies reported a less accurate perceived hand structure when the participants had non-informative visual cues, i.e., they could look at the tabletop covering their hand and at their localization judgments (visual condition), compared to when participants performed the task while blindfolded, relying only on somatic cues [6, 7]. Authors suggested that non-informative visual information may interfere with somatosensory processing, in line with previous evidence [53, 54], and that participants strongly rely on non-informative vision, as the primary sensory modality to organize behavior [6]. Along these lines, it is plausible that non-informative visual cues, by interfering with somatosensory processing, increased the somatic noise and thus the magnitude of a prototype bias. This could be tested by determining whether the increased finger underestimation in the visual condition is driven by a greater and/or more consistent proximal error of the fingertips towards the center of the hand or finger. In other words, a higher interference between vision and somatosensation may increase the weight of the prototype bias on the localization judgements, since this source of bias seems to generally weigh more in noisier conditions [41, 42].

In certain conditions, vision of the set-up seems to interfere with somatic processing. However, certain visual cues might actually inform about the location and actual physical size of the participant’s hand. For instance, by varying the surface size of the concealing surface, i.e., the panel, box or tabletop covering the participant’s hand during localization judgments, experimenters could indirectly cue the participants about the location and size of their hand, and thus modulate the error components and consistency. Specifically, by reducing the surface size, the magnitude of the overlap bias towards the shoulder might be reduced, whereas the consistency of the localization judgements might increase. This would be useful to better isolate errors that are specific to the fingertip and knuckle proprioceptive localization, and to test whether the overlap bias is actually the major factor producing the apparent hand width overestimation, a possibility that has been discussed above.

Another interesting avenue for future work would be comparing the error components and its consistency across populations to gain further insight about the nature of similarities and differences between groups. Starting from a classic comparison in experimental psychology, it would be interesting to explore how the localization errors differ between genders. Gender differences in hand representations are debated. A previous study found greater finger length underestimation and reduced hand width overestimation in male participants compared to female participants [20]. However, these differences were minimized when the participants’ actual hand size was modeled as a covariate, suggesting that they are likely to be related to the physical properties of the hand, rather than to a gender asymmetry, per se [8]. In detail, authors put forward the possibility that the hand structure may be distorted toward a prototypical hand [8]. In terms of localization errors, this would translate into a bias of the perceived locations of the participant’s *own* hand landmarks toward the prototypical locations of those landmarks in *a* hand; therefore it would lead to the prediction that the error magnitude of different landmarks varies in function of the physical hand size and therefore between genders. In contrast, we would predict that differences across genders could not be (solely) explained by a different pattern of angle consistency across different landmarks. While we did not find significant correlations between the physical hand size and localization error components and consistency in our sample (**S1 File**), future research should test those predictions on larger and more homogeneous samples, in order to further explore gender-specific differences in the LT. The hypothesis that biases related to spatial memory play a role in the LT, addressed in previous work [8] and earlier in this discussion, is promising, but needs to be further investigated and established. For instance, it should be tested whether those biases are toward a prototypical hand, or toward the center of the participant’s own hand or finger, i.e., the center of the categorical space, predominantly influencing judgments in conditions of increased sensory noise, in line with experiments on spatial memory and tactile localization [40, 42]. Furthermore, it is important to understand whether biases related to spatial memory modulate the localization of the fingertips, as we suggested above, or of both the fingertips and knuckles, as suggested previously [8]. In the latter case, however, accounting for the asymmetry that we described for the medio-lateral errors involving the knuckles might be more challenging.

With the present work, we provided some evidence that both domain-general and body-specific biases are likely to affect the proprioceptive localization of hand landmarks, and thus the distortions of the perceived hand structure. In their seminal study [3], Longo & Haggard (2010) found that participants tended to underestimate their finger length and overestimate their hand width, both when their hand was placed in the canonical upright position (-18.2% finger length underestimation, 63.5% hand width overestimation), and when their hand was rotated of 90º clockwise relative to the torso (-16.9% finger length underestimation, 48.8% hand width overestimation, but see [55, 56]: 2.3% and 7.39% hand width overestimation). This finding led them to conclude that domain-general spatial biases are unlikely to affect the finger length underestimation and the hand width overestimation, since only body-specific biases would be maintained once the hand is rotated. However, this finding is not incompatible with the influence of a prototype bias on fingertip localization, since, when the (left) hand is rotated, a prototype bias would lead to large and consistent lateral errors, oriented towards the center of the categorical space, i.e., the hand or the finger itself, producing—or simply enhancing—the finger length underestimation. Furthermore, their finding does not rule out that the hand width overestimation may be explained by medio-lateral (or proximo-distal) asymmetries of the overlap bias affecting the hand landmark localization. Indeed, the PCA detected a major source of proprioceptive bias (PC1) affecting the lateral landmarks, either fingertips or knuckles, more than the medial ones. Once this source of bias had been removed from the data, the hand width overestimation was minimized (**Fig 6B**). We argued that this proprioceptive bias might be analogous to the overlap bias previously reported [47, 48], suggesting that the hand width overestimation may not reflect a metric bias affecting the perceived hand dimensions, rather the effect of the differential weight of the overlap bias across the medio-lateral or proximo-distal axis. The analysis of the localization errors in the rotated posture would be crucial to shed light on the matter. In principle, it should be possible to understand whether the PC1, instead of the overlap bias, reflects a hand overestimation bias, by which the little knuckle is systematically mislocated more laterally (or distally) compared to the index finger. This seems unlikely, since the PC1 represents a source of error that similarly affected both fingertips and knuckles. However, if this is the case, such hand overestimation bias would be reflected by a 90º-rotated pattern of increasing index-to-little loadings affecting the distal errors (or decreasing loadings affecting the proximal errors, assuming that an overlap bias would anyway be present, see **S4 Fig in S1 File**), associated with the PC1 of the PCA on the proximo-distal errors. In contrast, if the PC1 represents the overlap bias, in the rotated posture we would expect similar loadings affecting the proximo-distal errors across the knuckles, or, even, increasing index-to-little loadings affecting the lateral errors (see **S4 Fig in S1 File**). Indeed, the medio-lateral component of the overlap bias is greater for localization judgements of landmarks located more distally from the body, as reported in previous investigations [48]. This last scenario would be compatible with the presence of a significant overestimation of the hand width in the rotated posture, which has been reported previously [3], but not systematically replicated [55, 56].

## Conclusions

To resume, for the first time we characterized the spatial and temporal features of the proprioceptive errors arising from the hand LT, previously used to measure the perceived hand structure [3]. We found greater proprioceptive errors for the fingertips compared to the knuckles (**Figs 1** and **2**), possibly reflecting a noisier proprioceptive input for the former compared to the latter. The perceived position of the knuckles might be indeed better informed by somatic signals coming from the muscle spindles. However, a poor proprioceptive signal per se does not account for the higher consistency in the error direction for the fingertips compared to the knuckles. The fingertips were indeed systematically displaced proximally and laterally (**Figs 3B** and **4B**). We argue that the localization of the fingertips does not uniquely rely on a poor proprioceptive signal, instead it may rely on the integration with other sources of information. We identified two (not mutually exclusive) mechanisms that are compatible with our results. First, to minimize the error in condition of poor proprioceptive signal, a prototype bias could have affected the judgments, similarly to what occurs in other domains [40, 42]. Concretely, participants misplaced the fingertips towards the center of the categorical space, which in this case could be the hand, or the finger itself. Second, in the condition of poor proprioceptive signal, participants may have relied on an underestimated representation of their finger length, which has been described in visual-based body representation tasks as well [44]. Further studies need to explore the weight of domain-general and body-specific sources of information affecting the fingertip misplacement.

We also found that information pertaining to the body structural description [45, 46] might also influence the localization of some hand landmarks. Indeed, knuckles that are closer to the physical boundaries of the hand, whose localization is thus informed by general knowledge about the body (e.g.: the thumb knuckle is next to the physical boundary of the hand), are more similarly misplaced across participants (**Fig 4C**), than knuckles whose localization is less informed by structural information (e.g.: the middle finger knuckle). A similar mechanism could have also contributed to the proximal misplacement of the fingertips. Importantly, those sources of information might be very relevant for the localization of body landmarks (besides the fingertips) when proprioceptive afference is reduced or extremely noisy as a result of somatosensory damage.

Furthermore, we showed that the localization judgments of all the landmarks are similarly affected by a misplacement towards the shoulder of origin, coherently with the overlap effect described in the literature [47, 48], and are drifted proximally over time, probably reflecting a spontaneous proprioceptive drift [49, 50, 52]. The overlap effect systematically influenced participant judgments, resulting in a relatively high between- and within-subjects angle consistency, in line with what reported by previous work [47].

Importantly, we argued that the overlap effect is represented by the PC1 extracted by the PCA on the proximo-distal and medio-lateral errors (**Fig 5**). The effect of the PC1 in the medio-lateral axis increased from the index to the little finger, enhancing the pattern of increasing index-to-little lateral error that underlies the hand width overestimation (**Figs 6** and **7**, simulation 6). If the PC1 represents the overlap bias, the hand width overestimation might therefore be a consequence of a greater overlap bias affecting the lateral compared to the medial landmarks.

To conclude, our work offers an original perspective to the investigation of proprioceptive and spatial biases in body perception, by proposing an alternative analytical approach (documented in the freely accessible code; [35]), which can be complementary to the more traditional analysis of data collected from the LT. While the pattern of distortions affecting the metric representation of the hand has been attributed to its cortical representation in the somatosensory cortex [57], we did not find evidence of a one-to-one relationship between proprioceptive accuracy and perceived hand distortions. Instead, our results suggest that such pattern of distortions might be the result of a much more complex combination of different factors, either related or unrelated to the hand cortical representation. These factors may include actual metric biases affecting the perceived finger length, which might be related to their cortical representation [57], and other factors pertaining to structural information about the body in general (body structural description), proprioceptive (overlap effect) and spatial memory (prototype) biases. In particular, the hand width overestimation might be the result of a differential weight of the overlap bias on the medio-lateral axis, which might be compatible with the results of previous investigations [58], rather than the result of shape anisotropies of the receptive field sizes, as suggested by previous work [57].

Importantly, we provided some hints in order to exploit in future research the analysis of proprioceptive localization errors to gain important insights on the nature of the biases affecting the hand metric representation.

## Supporting information

S1 File (DOCX)
