# Supplementary material for: Proprioceptive errors in the localization of hand landmarks: What can be learnt about the hand metric representation?
Source: PLoS One. 2020 Jul 31;15(7):e0236416. doi: 10.1371/journal.pone.0236416 (PMC7394425; doi:10.1371/journal.pone.0236416)
Supplement: S1 File — (DOCX) [file pone.0236416.s001.docx]

**Proprioceptive errors in the localization of hand landmarks:**

**what can be learnt about the hand metric representation?**

Valeria Peviani^1,2,*^ & Gabriella Bottini^1,3,4^

^1^ Department of Neuroscience, Max Planck Institute for Empirical Aesthetics, [Grüneburgweg 14, 60322, Frankfurt am Main](https://maps.google.com/?q=Gr%C3%BCneburgweg+14+,++60322+-+Frankfurt+am+Main&entry=gmail&source=g), Germany

^2^ Department of Brain and Behavioural Sciences, University of Pavia, Via Bassi, 21, 27100, Pavia, Italy

^3^ Cognitive Neuropsychology Center, ASST Grande Ospedale Metropolitano Niguarda, Piazza dell’Ospedale Maggiore 3, 20162, Milan, Italy

^4^ NeuroMI, Milan Center for Neuroscience

*** Corresponding Author**

Email address: [valeria-carmen.peviani@ae.mpg.de](mailto:valeria-carmen.peviani@ae.mpg.de)

**SUPPORTING INFORMATION**

**1) Combining data from two experiments with different number of trials**

The two datasets we analyzed in this work involved a very different number of trials (experiment 1: 400 trials, experiment 2: 160 trials). To be able to exploit the entire datasets and explore how the localization errors varied over the experiments, we decided to normalize the trial number (1 to 400 and 1 to 160 are normalized to cover a range within 1 and 100) and study its effect on error components and consistency. However, given their different length in time, the effect of normalized trial may be different between experiments, for example due to increased fatigue or attentional drop. We explored this possibility by running the additional analyses that follow.

To this first aim, we fitted a linear mixed-effects model with experiment and normalized trial as predictors, and either the medio-lateral or the proximo-distal systematic error component as dependent variable. The experiment-by-normalized trial interaction is informative on whether the effect of normalized trial varies across experiments. Regarding the medio-lateral component, we found a main effect of experiment (F(1,11349) = 8.429, p = .003), and no effect of normalized trial (F(1,11349) = 2.357, p = .125), nor interaction (F(1,11349) = 0.240, p = .624). In detail, medio-lateral errors in experiment 2 were generally higher (lower negative values) than in experiment 1, but the two experiments did not differ in how the errors varied over time (**Figure S1A**). Regarding the proximo-distal component, we found a main effect of normalized trial (F(1,11349) = 5.130, p = .023), no effect of experiment (F(1,11349) = 3.135, p = .076) and a significant interaction (F(1,11349) = 4.855, p = .027. In particular, the slope describing the effect of the normalized trial on the proximo-distal errors was steeper for experiment 2 (β = -0.020) than for experiment 1 (β = -0.007). By observing the scatterplot (**Figure S1B**), it looks like the interaction is driven by a difference in the errors made at the beginning on the task, while errors progressively become similar
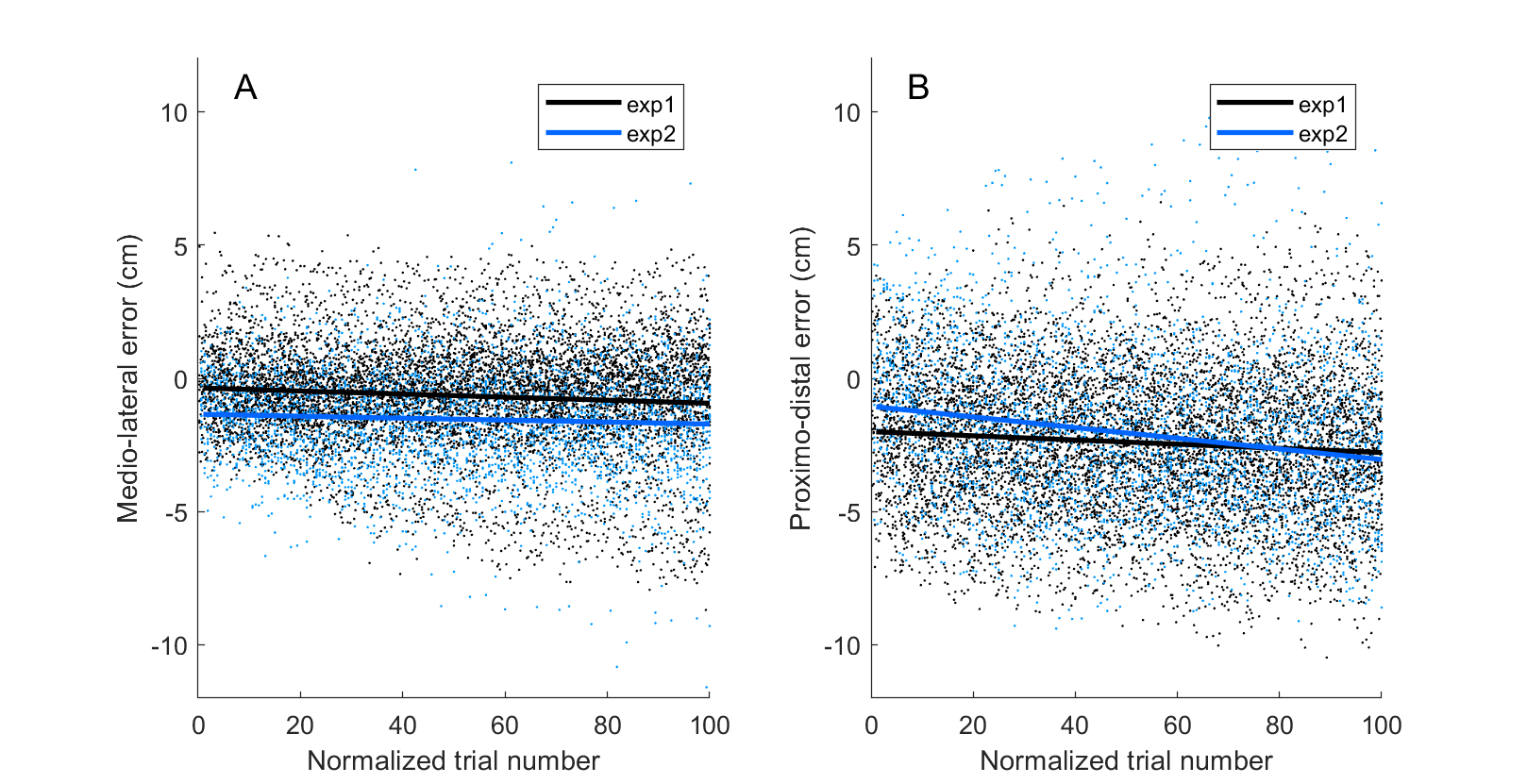
between tasks.

***Figure S1.*** *Slopes obtained from the linear mixed-effects models describing the effect of the normalized trial number on medio-lateral* ***(A)*** *and proximo-distal* ***(B)*** *errors are represented for experiment 1 (black) and experiment 2 (blue).*

This was confirmed by an analysis that considered only the first 35 (‘initial’ trials) and the last 30 trials (‘final’ trials) of each task. The asymmetry between the number of initial and final trials considered is due to the higher number of outlier observations within the initial trials. We fitted a linear mixed-effects model with experiment and trial cluster (initial vs. final) to predict the medio-lateral and proximo-distal error components. As expected, we found significant main effect of experiment (F(1,2557) = 136.850, p <.001) and trial cluster (F(1,2557) = 36.020) but no interaction (F(1,2557) = 1.081, p = .298) on the medio-lateral errors. Furthermore, we found significant effects of experiment (F(1,2557) = 10.504, p = .001), trial cluster (F(1,2557) = 130.937, p <.001) and a significant interaction (F(1,2557) = 18.730, p <.001) on the proximo-distal errors. Importantly, the post-hoc comparisons revealed a significant difference between the experiments only for the initial trials, with greater errors (lower negative values) for experiment 2 compared to 1 (initial trials: mean difference (exp1 - exp2) = -.831 ± .156, p <.001; initial trials: mean difference (exp1 - exp2) = .119 ± .155, p = .441).

Taken together, these results suggest that the medio-lateral errors are not affected by the number of trials included the task. Regarding the proximo-distal errors, the outcome is more uncertain. We confirmed that the effect of normalized trial is different between experiments, with greater proximo-distal errors in experiment 2 at the beginning of the experiment, which also decreased faster (steeper slope) than the error of experiment 1, reaching similar values by the end of the experiment. To better understand whether difference is likely to be due to the different number of trials, we ran the same analysis, i.e., the linear mixed-effects model with experiment and trial number as predictors and proximo-distal errors as dependent variable, considering only the first 160 trials of each experiment and the raw trial number, rather than the normalized trial, as predictor. Indeed, if the effect is driven by the different number of trials, it should not be present when these two equally-large subsets of data are considered. The results of this analysis were similar to the previous ones, with a significant experiment-by-trial interaction F(1,6474) = 6.681, p = .009) and no significant main effects of trial (F(1,6474) = 2.915, p = .087) and experiment (F(1,6474) = 2.017, p = .155). Again, a steeper slope was observed in experiment 2 (β = -0.009 ± 0.003) compared to experiment 1 (β = -0.003 ± 0.002), see **Figure S2**.

This last result led us to conclude that the different slopes describing the effect of trial number on the proximo-distal errors is not due to a difference in the number of trials between experiments. This means that other factors may have played a role, such as interindividual variability, or slight variations of the experimental procedures and environment. For instance, half of the participants of experiment 1 were administered with a proprioceptive matching task, before the Localization Task (LT). As another example, participants in experiment 2 were ready to commit for a much longer period of time (three two-hours sessions instead of one).


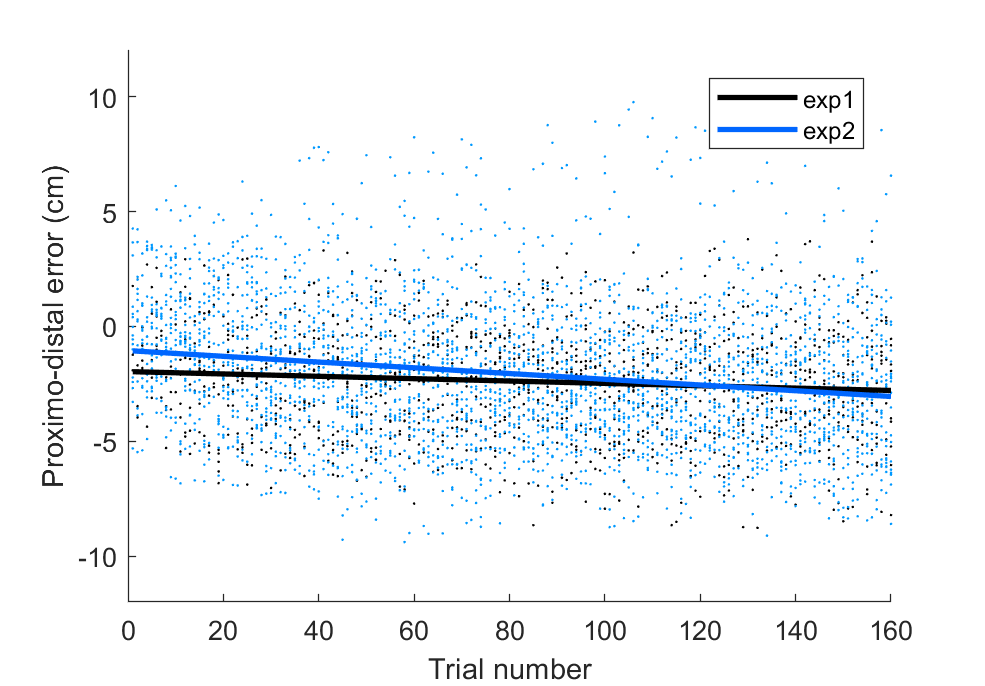


***Figure S2.*** *The slope obtained from the linear mixed-effects model describing the effect of the raw trial number (1 to 160) on proximo-distal errors are represented for experiment 1 (black) and experiment 2 (blue).*

**2) Simulation procedure**

We aimed at showing that similar patterns of metric biases affecting the perceived hand structure may be underlined by very different patterns of localization biases. To this aim, we simulated data of 30 participants with 200 trials each, equally distributed among the ten hand landmarks, starting from three sets of parameters extracted from our real data, and compared different scenarios resulting from the change of these parameters.

Given a set of localization errors (thin vectors in **Figure S3A**), the x and y components (dashed red and blue segments in **Figure S3A**) of the average error vector (bold vector in **Figure S3A**), which represents the shift from the real to the perceived position of a certain landmark, and thus contributes to determine the perceived distance from that landmark to the other ones, can be well-approximated as follows:

$$\vec{v_{x}}\cong\left| \vec{v} \right| \times\left| \vec{r} \right| \times\cos\theta$$

$$\vec{v_{y}}\cong\left| \vec{v} \right| \times\left| \vec{r} \right| \times\sin\theta$$

Where $\left| \vec{v} \right|$ is the absolute magnitude, i.e., the length, of the average error vector (**Figure S3A**),$\left| \vec{r} \right|$ is the absolute magnitude of the average error vector in the polar space, corresponding to the angle consistency and ranging 0 to 1 (**Figure S3B**), and $\theta$ is the angle of such vector (most frequent error direction, **Figure S3B**).


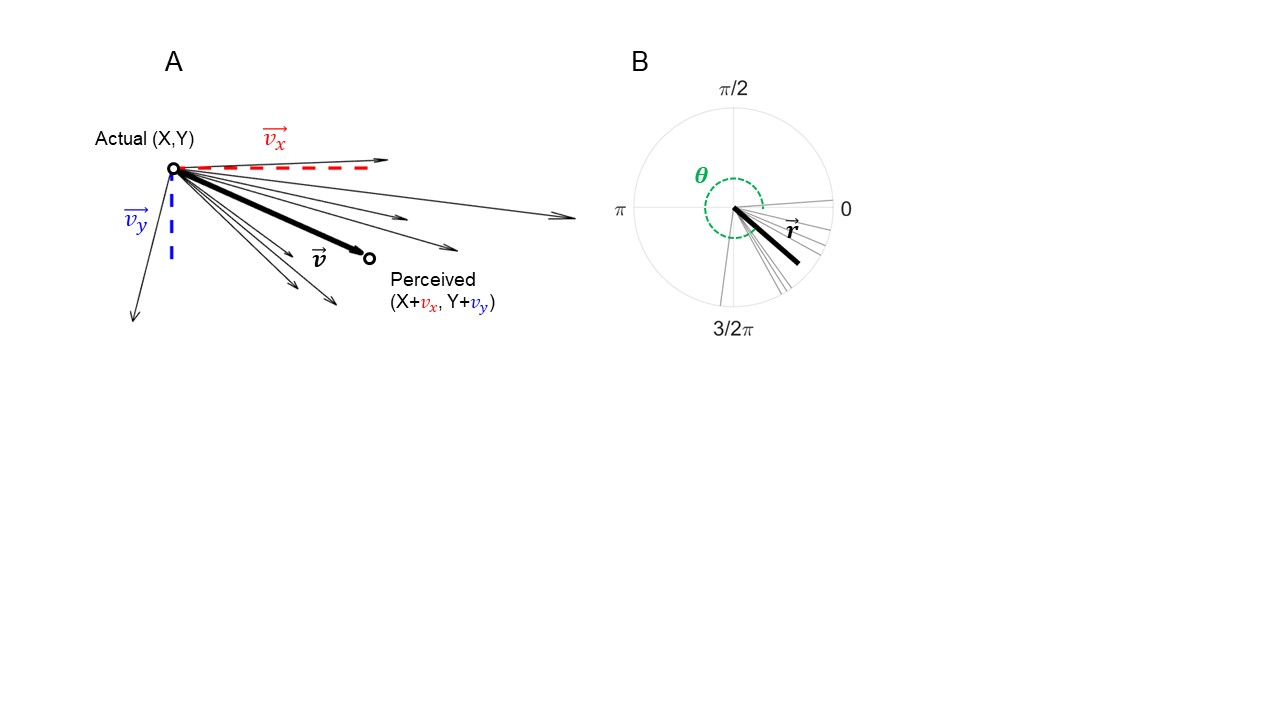


***Figure S3.*** ***(A)*** *Example of a landmark localization bias (*$\vec{v}\boldsymbol{)}$ *obtained by averaging the x and y components of several error vectors. The localization bias determines the landmark perceived position.* ***(B)*** *The same error vectors are represented in a polar space, in which their direction is maintained but their magnitude, i.e., their length, equals 1. Their x and y components are averaged to obtain the average error vector (*$\vec{r}).$ *The magnitude of this vector (*$\left| \vec{r} \right|)$ *represents the angle consistency, while* $\theta$ *represents the most frequent error direction.*

For each landmark, we thus extracted the magnitude of the average error vector by applying Pythagorean theorem on its x and y components, the median within-subject angle consistency (as described in the method) and the median error direction. Regarding this last parameter, while the paper reports the median of our participants’ most frequent error directions, regardless of the landmark, here we extracted the most frequent error direction for each participant and landmark. We simulated inter-individual and -landmark variability by adding noise randomly sampled from a normal distribution to the absolute magnitude of the error vector, the most frequent error direction and angle consistency. For the error magnitude, we followed a similar procedure to add inter-trial noise. The variation of this noise is reported as a standard deviation in **Table S1**.

| **Hand landmark** | **Absolute error magnitude (cm),** $\left\vert\vec{\boldsymbol{v}} \right\vert$ | **Angle consistency,** $\left\vert\vec{\boldsymbol{r}} \right\vert$ | **Most frequent error direction (degrees),** $\boldsymbol{\theta}$ |
| --- | --- | --- | --- |
| ***Thumb fingertip*** | 3.775 ± 0.240 | 0.985 ± 0.030 | 245.087 ± 2.451 |
| ***Index fingertip*** | 4.252 ± 0.240 | 0.988 ± 0.030 | 256.869 ± 2.569 |
| ***Middle fingertip*** | 4.104 ± 0.240 | 0.990 ± 0.030 | 257.908 ± 2.579 |
| ***Ring fingertip*** | 4.461 ± 0.240 | 0.985 ± 0.030 | 238.207± 2.382 |
| ***Little fingertip*** | 4.148 ± 0.240 | 0.986 ± 0.030 | 244.122 ± 2.441 |
| ***Thumb knuckle*** | 1.259 ± 0.240 | 0.926 ± 0.030 | 232.797 ± 2.328 |
| ***Index knuckle*** | 0.954 ± 0.240 | 0.924 ± 0.030 | 248.638 ± 2.486 |
| ***Middle knuckle*** | 0.327 ± 0.240 | 0.898 ± 0.030 | 188.902 ± 1.889 |
| ***Ring knuckle*** | 1.116 ± 0.240 | 0.891 ± 0.030 | 196.107 ± 1.961 |
| ***Little knuckle*** | 1.508 ± 0.240 | 0.900 ± 0.030 | 209.088 ± 2.091 |

***Table S1.*** *The parameters used to obtain the simulated data. Means and medians (obtained from the real data) are reported for the absolute magnitude of the error and for the angle consistency and direction, respectively. Their variation represents the standard deviation of the normal distribution from which the inter-individual, -trial and -landmark noise has been sampled.*

For each simulated participant and landmark we were thus able to estimate the x and y components of the average error vectors, add them to the real hand landmark positions (randomly sampled from our dataset) to compute the perceived hand positions, and thus distances between hand landmarks. As indexes of the perceived hand structure, we considered the estimation ratio (ER) between the perceived and actual hand width (distance between index and little knuckles) and between the perceived and actual mean finger length (average distance between each finger and the relative knuckle). The outcome of the average perceived hand structure and of the simulated data is represented in **Figure 7** (main text, top-left panel). This is similar to the perceived hand structure of our real data (means and standard deviations of finger length ER: 0.578 ± 0.092; hand width ER: 1.308 ± 0.274), although the latter is characterized by a greater hand width overestimation, as detected through t-tests (finger length: t(71) = 0.989, p = .326; hand width ER: t(71) = -3.263, p = .002). This difference might be due to the fact that the variability might differ across individual, landmarks and trials, a parameter that we did not model in our simulated data. Afterwards, we varied one or more initial parameters (**Table S2**) to obtain seven patterns of hand structure, with varying mean finger length and hand width (**Figure 7,** main text). We used t-tests to compare the resulting ERs. Regarding the finger length ERs, a significant difference (p<.001) was detected between: datasets 1 and 3, 1 and 4, 2 and 3, 2 and 4. Regarding the hand width ER, a significant difference (p<.001) was detected between: datasets 1 and 6, 1 and 7, 5 and 6, 5 and 7. All the other contrasts were not statistically significant.

| **Outcome** | **Params.** | **Fingers** | **Knuckles** |
| --- | --- | --- | --- |
| **1** | $\left\vert\vec{\boldsymbol{v}} \right\vert$  $\left\vert\vec{\boldsymbol{r}} \right\vert$  $\boldsymbol{\theta}$ | 4.146 ± 0.240  0.965 ± 0.030  246.795 ± 2.484 | 1.023 ± 0.240  0.887 ± 0.030  209.217 ± 2.151 |
| **2** | $\left\vert\vec{\boldsymbol{v}} \right\vert$  $\left\vert\vec{\boldsymbol{r}} \right\vert$  $\boldsymbol{\theta}$ | **5.038** ± 0.240  **0.801** ± 0.030  246.049 ± 2.484 | 1.019 ± 0.240  0.883 ± 0.030  208.898 ± 2.151 |
| **3** | $\left\vert\vec{\boldsymbol{v}} \right\vert$  $\left\vert\vec{\boldsymbol{r}} \right\vert$  $\boldsymbol{\theta}$ | 4.152 ± 0.240  0.968 ± 0.030  246.614 ± 2.484 | **1.728** ± 0.240  0.885 ± 0.030  209.554 ± 2.151 |
| **4** | $\left\vert\vec{\boldsymbol{v}} \right\vert$  $\left\vert\vec{\boldsymbol{r}} \right\vert$  $\boldsymbol{\theta}$ | 4.157 ± 0.240  **0.803** ± 0.030  246.624 ± 2.484 | 1.002 ± 0.240  0.884 ± 0.030  208.507 ± 2.151 |
| **5** | $\left\vert\vec{\boldsymbol{v}} \right\vert$  $\left\vert\vec{\boldsymbol{r}} \right\vert$  $\boldsymbol{\theta}$ | 4.143 ± 0.240  0.970 ± 0.030  245.906 ± 2.484 | 1.070 ± 0.240  0.463 ± 0.030  **232.774 ± 2.417** |
| **6** | $\left\vert\vec{\boldsymbol{v}} \right\vert$  $\left\vert\vec{\boldsymbol{r}} \right\vert$  $\boldsymbol{\theta}$ | 4.098 ± 0.240  0.964 ± 0.030  245.917 ± 2.484 | **1.406** ± 0.240  0.884 ± 0.030  209.115 ± 2.151 |
| **7** | $\left\vert\vec{\boldsymbol{v}} \right\vert$  $\left\vert\vec{\boldsymbol{r}} \right\vert$  $\boldsymbol{\theta}$ | 4.120 ± 0.240  0.969 ± 0.030  246.743 ± 2.484 | 1.027 ± 0.240  0.885 ± 0.030  **232.462 ± 2.417** |

***Table S2.*** *Combinations of initial parameters (*$\left| \vec{v} \right|$*: absolute error magnitude;* $\left| \vec{r} \right|$*: angle consistency;* $\theta$*: most frequent error direction) used to obtain the seven simulations which produced different hand perceived structures (****Figure 7,*** *main text). Variations of the parameters relative to the first simulation are highlighted in bold. Means are reported for the absolute error magnitude, whereas medians are reported for the angle consistency and most frequent error direction. The variation (±) indicates the standard deviation of the normal distribution from which the random noise added to the data was sampled from.*

**3) Effect of physical size**

We explored the relation between physical hand size (average finger length and hand width) and localization error parameters (average magnitude of medio-lateral and medio-lateral errors and within-subjects angle consistency) through correlational analyses (Spearman’s r). None of the correlations was significant. See **Table S3**.

| **Physical hand size** | Correlated with: | **Proximo-distal error** | **Medio-lateral error** | **Within-subject angle consistency** |
| --- | --- | --- | --- | --- |
| ***Average finger length*** |  | r_s_ = 0.139, p = .376 | r_s_ = 0.039, p = .799 | r_s_ = -0.188, p = .227 |
| ***Hand width*** |  | r_s_ = 0.091, p = .561 | r_s_ = -0.003, p = .983 | r_s_ = -0.068, p = .665 |

***Table S3.*** *Spearman’s correlation values between the hand physical dimensions (average finger length and hand width) and localization error parameters (proximo-distal and medio-lateral errors, within-subject angle consistency) are reported.*

**4) Possible PCA scenarios on proximo-distal and medio-lateral errors in LT with 90°-rotated hand posture**

Performing a PCA on the medio-lateral and proximo-distal errors measured through the LT with the hand in the 90°-rotated posture would be useful to test whether the medio-lateral PC1 (first principal component) reported in the main text (**Figure 5**) represents the hand overestimation bias (greater lateral misplacement of the little finger knuckle compared to the index finger) knuckle or the overlap bias. In the first case, the PCA should detect a 90°-rotated index-to-finger increasing loading (thus, error) pattern, as showed by the PCA vectors of the first scenario depicted in **Figure S4**. Assuming that an overlap bias may still affect the localization, the loadings and errors would increase from the little to the index finger in the proximal axis (second scenario). Instead, if PC1 represents the overlap bias, we would expect an index-to-little increase of the loadings (and of the errors) on the lateral axis (third scenario), which would explain the hand width overestimation observed in the 90°-rotated LT (3). Indeed, the medio-lateral component of the overlap bias tends to increase when it pertains to localization judgement for landmarks that are further away from the body (48).

**
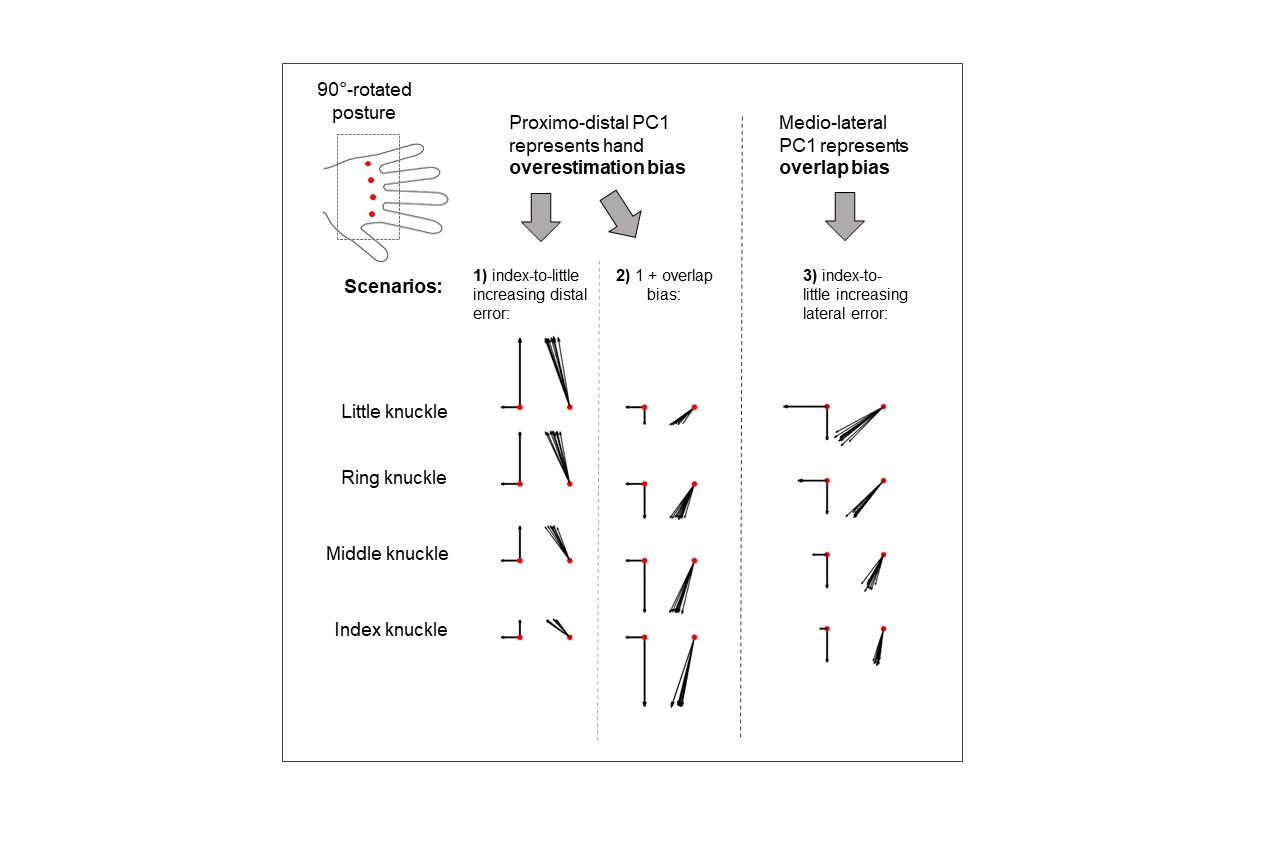
**

***Figure S4.*** *Three scenarios regarding the possible results of a PCA on the medio-lateral and proximo-distal errors for knuckle localization during the LT with the hand in 90°-rotated posture. For each scenario, the PC1 vectors and associated localization errors are depicted.*
